# Subcutaneous platelet-rich plasma and topical graphitic carbon nitride in a lanolin carrier enhance healing of surgically induced cutaneous wounds in dogs

**DOI:** 10.1186/s12917-026-05739-7

**Published:** 2026-07-23

**Authors:** Mona N. Wafy, Elham A. Hassan, Samar Saeed, Marwa S. Khattab, Huda O. AbuBakr, Ashraf M. Abu-Seida

**Affiliations:** 1 Department of Surgery, Anesthesiology and Radiology- Faculty of Veterinary Medicine, Cairo University, Giza, 12211 Egypt; 2 National Institute of Laser Enhanced Sciences, Cairo University, Giza, 12613 Egypt; 3 Department of Pathology, Faculty of Veterinary Medicine, Cairo University, Giza, 12211 Egypt; 4 Department of Biochemistry and Molecular Biology- Faculty of Veterinary Medicine, Cairo University, Giza, 12211 Egypt; 5 Department of Biochemistry, Faculty of Veterinary Medicine, Egyptian Chinese University, Cairo, Egypt; 6 Animal Research Facility, Galala University, Galala City, Suez, Egypt

**Keywords:** Dogs, Graphitic Carbon Nitride, Wound Healing, Nanomaterials

## Abstract

**Background:**

Graphitic carbon nitride nanoparticles (g-C3N4) are a promising material in wound healing; however, their effects and potential synergy with PRP (platelet-rich plasma) remain unexplored. This study evaluates the single and synergistic effects of PRP injection and g-C3N4 nanoparticle ointment on wound healing in dogs. Six full-thick skin wounds per group (a total of 6 groups) were surgically created on the chest region of six adult mongrel dogs, with each dog having three bilateral chest wounds. Wounds were treated with either normal saline, lanolin dressings, PRP infiltration, PRP infiltration combined with lanolin dressings, g-C3N4 NPs ointment, or PRP infiltration with g-C3N4 NPs application. Healing progression was monitored, with the evaluation of epithelialization and wound contraction. Total antioxidant capacity (TAC), malondialdehyde (MDA), and PDGFβ concentration were measured in wound fluid. Matrix extracellular phosphoglycoprotien (MEPE) and transforming growth factor beta (TGF-β) gene expressions were assessed in skin biopsies at 0, 5, 10 and 20 days. Wound histopathology and tumor necrosis factor alpha (TNF-α) immunohistochemistry (IHC) were performed.

**Results:**

Both PRP and PRP–g-C3N4 nanoparticles significantly enhanced healing. PRP alone greatly improved regeneration, yet with increased oxidative stress. These included maximal healing, epithelialization, high levels of gene expression, efficient inflammation control and well-organised granulation tissue and collagen bundles. PRP–g-C3N4 NPs offered a more balanced profile, exhibiting minimal lipid peroxidation, superior antioxidant preservation, and moderate but sustained regeneration.

**Conclusion:**

Although PRP is excellent for quick tissue repair, PRP–g-C3N4 NPs might be better suited for situations requiring oxidative stability and controlled inflammation.

## Introduction

Cutaneous wounds are among the most common clinical conditions encountered in veterinary practice, particularly in dogs, and may result from trauma, surgery, infection, burns, or pressure-related injuries. Wound repair is a complex and coordinated biological process involving hemostasis, inflammation, proliferation, and tissue remodeling. Any disturbance in the timing or progression of these phases may delay healing and increase the risk of infection, chronic inflammation, and poor scar formation [1]. Although canine wounds generally exhibit a relatively rapid and centrally directed healing response, advanced therapeutic approaches, including platelet-rich plasma (PRP) and specialized wound dressings, may further improve healing outcomes [2, 3].

Platelet-rich plasma is an autologous plasma preparation containing a concentrated number of platelets and biologically active growth factors. These include platelet-derived growth factor, vascular endothelial growth factor, and transforming growth factor-beta, which contribute to the regulation of inflammation, angiogenesis, fibroblast proliferation, collagen synthesis, and extracellular matrix remodeling [3–5]. PRP can be administered topically or through local subcutaneous infiltration around the wound margins. Previous studies in dogs have demonstrated that PRP improves wound contraction, epithelialization, collagen deposition, dermal organization, and the overall quality of newly formed tissue [6, 7]. Its autologous origin, relative affordability, and low risk of adverse immune reactions also support its potential clinical application in veterinary wound management.

Nanotechnology has introduced new possibilities for wound treatment through materials with distinctive physical, chemical, and biological properties at the nanoscale. Nanomaterials may improve tissue interaction, local bioavailability, sustained therapeutic activity, and antimicrobial protection compared with conventional topical agents and dressings [8–10]. These features are particularly relevant in wounds complicated by persistent inflammation, microbial contamination, or delayed tissue regeneration.

Graphitic carbon nitride (g-C₃N₄) is a metal-free polymeric semiconductor composed mainly of carbon and nitrogen. It has a two-dimensional layered structure and is characterized by chemical stability, biocompatibility, ease of synthesis, and photocatalytic activity [11–14]. In wound-care applications, g-C₃N₄ nanosheets have demonstrated antibacterial and antibiofilm effects through reactive oxygen species generation and direct interaction with bacterial membranes [15]. Moreover, g-C₃N₄-containing composite dressings have been reported to enhance fibroblast migration, collagen deposition, epithelialization, and wound closure, indicating that their effects may extend beyond microbial control to the promotion of tissue regeneration [16].

Biochemical and molecular biomarkers provide valuable complementary information for evaluating wound-healing progression. In the present study, total antioxidant capacity (TAC), malondialdehyde (MDA), PDGF-β, MEPE, TGF-β, and TNF-α were evaluated to reflect changes in oxidative balance, inflammatory activity, growth-factor signaling, extracellular matrix formation, and tissue remodeling. Collectively, these biomarkers, together with clinical and histopathological findings, can provide a more comprehensive assessment of the effectiveness and progression of wound healing [17].

Despite the reported regenerative effects of PRP and the promising biological properties of g-C₃N₄-based materials, their combined influence on cutaneous wound healing has not been investigated in dogs. Therefore, the present study aimed to evaluate the individual and combined effects of a single local PRP infiltration and daily topical application of g-C₃N₄ nanoparticle ointment on surgically induced cutaneous wound healing in dogs.

Our study aims to evaluate the impact of daily application of g-C3N4 NPs ointment and a single local PRP infiltration and their combination on the healing process of cutaneous wounds in dogs.

## Materials and methods

### Ethical approval

The current study was approved by the Institutional Animal Use and Care Committee at the Faculty of Veterinary Medicine, Cairo University, Egypt (Approval number: Vet CU 08072023704). Additionally, the Animal Research: Reporting in Vivo Experiments (ARRIVE) guidelines were adhered to.

### Experimental animals

Six healthy adult mongrel dogs, aged between one and two years and weighing between 15 and 20 kg were commercially bought from Al-Fahad Trading Company for Animals (Abu-Rawash, Giza, Egypt) and used in this study. Before the experiment commenced, each dog underwent a thorough medical evaluation, including a complete blood count and serum biochemistry analysis. The dogs were housed individually in kennels measuring 1.5 m × 2.5 m × 3 m and were given a two-week acclimatization period to adjust to their new environment and diet. They were maintained under standard conditions, including a 12-hour light/dark cycle, a temperature of 25 ± 2 °C, humidity levels of 60 ± 5%, and adequate ventilation. Fresh water was provided adlibtum, and they were fed commercial maintenance dry food twice daily. These animals were obtained commercially from Al-Fahad Trading Company for Animals, Giza, Egypt.

### Study design

The six dogs each received three bilateral full-thickness skin wounds on their chests, totalling 36 wounds. These wounds were randomly assigned to six treatment groups (six wounds per group) as follows:

- Group 1 (Control group): Wounds were dressed with normal saline.
- Group 2 (Lanolin group): Wounds were dressed daily with lanolin only, which served as the carrier for g-C3N4 NPs.
- Group 3 (PRP group): Wounds were treated with a single local injection of autologous PRP.
- Group 4 (PRP-Lanolin group): Wounds received a single PRP injection followed by daily dressing with lanolin.
- Group 5 (g-C3N4 NPs group): Wounds were treated daily with an ointment containing g-C3N4 NPs carried in lanolin.
- Group 6 (PRP-g-C3N4 Nps group): Wounds received a single PRP injection and were dressed daily with g-C3N4 NPs ointment.

### Preparation of g-C3N4 NPs

A quartz tube with an inner diameter of 25 mm and a length of 1000 mm was used as the reaction chamber. Analytical-grade melamine powder (3 g) was compressed into a cylindrical column and placed at the center of the quartz tube. The chamber was then evacuated to a pressure below 1 Pa (approximately 10⁻² Torr). To ensure complete reaction, the initial materials were heated in an electric furnace at a controlled rate reaching 800 °C, where the temperature was maintained for 2 h under vacuum conditions. The quartz tube was then allowed to cool naturally to ambient temperature. The yellow powder that had formed on the inner walls of the quartz tube was then collected. To improve crystallization, the collected powder was subjected to one additional thermal treatment following the same procedure, resulting in a total of two pyrolysis cycles. Finally, g-C₃N₄ was successfully synthesized [18].

### Characterization of g-C3N4 NPs

#### Scanning Electron Microscopy (SEM)

The scanning electron microscopy (SEM) images were captured using a ZEISS FE-SEM ULTRA Plus microscope, which was equipped with an energy-dispersive X-ray (EDX) analyzer. Additionally, a Philips CM20 microscope was utilized, operating at an accelerating voltage of 200 kV.

#### Fourier Transform Infrared Spectrophotometer (FTIR)

A Fourier Transform Infrared (FTIR) spectrophotometer (*Jasco FT/IR 460 Plus*,* Tokyo*,* Japan*), fitted with an Attenuated Total Reflection (ATR) cell, was utilized to examine the FTIR spectra of the g-C3N4 nanoparticles (NPs). The samples were initially compressed into pellets using potassium bromide (KBr) before being placed onto the ATR crystal. The FTIR spectra were then measured at room temperature, with a resolution of 4 cm⁻¹ and a scanning speed of 2 mm⁻¹ over a wavelength range of 500–4000 cm⁻¹.

#### X-Ray Diffraction (XRD)

Cu Ka radiation (k = 1.54186 A°) was used in XRD measurements using a Philips PW1710 X-ray diffractometer. With a step size of 0.020° 2 H, the XRD patterns were recorded from 5° to 80° 2 H, collecting 10 s per step.

### Preparation of g-C3N4 NPs ointment

To prepare the G-C3N4 NPs ointment, two grams of G-C3N4 nanoparticles were first dissolved in 8 mL of N-Methyl-2-pyrrolidone (NMP) solvent. The g-C3N4 NPs were transferred to an appropriate container, followed by the addition of NMP, and the mixture was sonicated to ensure complete dissolution. Separately, 90 g of lanolin were measured, placed in a glass container, and melted into a uniform liquid using a sonicator or water bath. Once both the melted lanolin and the g-C3N4 solution were ready, the lanolin was carefully incorporated into the g-C3N4 mixture and stirred vigorously to ensure even distribution of the nanomaterial within the lanolin base. The final mixture transformed into an ointment, which was then transferred into storage containers. To improve its texture, the containers were briefly frozen for approximately five minutes to aid solidification. The final product was stored in a cool, dry place, away from direct sunlight, to maintain its stability and effectiveness [19].

### Preparation of autologous PRP

A 10 mL whole blood sample was collected from the cephalic vein of each dog using a citrate-phosphate dextrose solution as an anticoagulant. The blood was then centrifuged at 1500 rpm for 10 min, resulting in three distinct layers. The upper plasma layer was carefully transferred to a new centrifuge tube and subjected to a second centrifugation at 3000 rpm for 20 min, separating it into two layers: platelet-poor plasma (PPP) at the top and platelet-rich plasma (PRP) at the bottom. To activate the PRP, calcium chloride (CaCl₂) was added at a ratio of 1:10 (0.1 mL CaCl₂ per 1 mL PRP) [7]. Immediately after activation, 1 mL of PRP was injected subcutaneously into multiple wound sites to prevent plasma from solidifying [20].

### Evaluation of PRP

A complete blood count (CBC) was performed for each dog. After generating PRP, the platelet count was determined from the PRP sample using a Mindray Vet BC 5000 analyzer (Mindray Animal Medical Technology Co., LTD, China). Additionally, the samples were analyzed for the presence of white blood cells (WBCs) and red blood cells (RBCs).

### Surgical procedures

The dogs were premedicated with 0.05 mg/kg atropine sulfate (*Atropine*^®^, Sunways Pvt. Ltd., Mumbai, India) administered subcutaneously, followed by 1 mg/kg xylazine HCl 2% (*Xylamed*^®^, Bimeda Animal Health, Dublin, Ireland) given intramuscularly. Anesthesia was induced with 10 mg/kg ketamine HCl 5% (*Ketalar*^®^, JHP Pharmaceuticals, Michigan, USA) intramuscularly, and maintenance was achieved through an intravenous dose of 25 mg/kg thiopental sodium 2.5% solution (*Thiopental Sodium*^®^, Livealth Biopharma Pvt. Ltd., Mumbai, India).

The dogs were placed in a sternal position, and the bilateral dorsal midline regions were prepared for aseptic surgery by clipping, shaving, and disinfecting with povidone-iodine solution. Three circular full-thickness skin wounds, each measuring 3 cm in diameter, were surgically created on both sides of the thorax, positioned 5 cm from the dorsal midline [21]. Hemostasis was controlled using sterile gauze tampons, and all wounds were covered with sterile dressing, gauze, and an elastic bandage. To prevent the dogs from interfering with the wounds, Elizabethan collars were applied.

For pain management, carprofen (4 mg/kg) was administered orally once daily for up to three days post-surgery. Wounds were dressed daily according to their assigned treatment group.

### Objective evaluation of wound healing

The diameter of each wound was initially measured using a digital caliper on day 0, with subsequent measurements taken on days 7, 14, and 21. At the same time points, macroscopic images of the wounds were captured using a digital camera, ensuring a standardized ruler was included in each image for calibration. Wound size was determined through standardized image analysis techniques, utilizing Digimizer software [22].

The wound healing process was monitored daily, documenting any visible changes such as inflammation, granulation tissue formation, and epithelialization. Additionally, observations related to pain, discomfort, or signs of infection in the dogs were recorded.

The percentage of wound healing and epithelialization in all groups was calculated using the following formulas:

$$\mathrm{Wound}\;\mathrm{size}\;\mathrm{percentage}\;\left(\%\mathrm{WS})\;\mathrm{at}\;\mathrm{day}\;(\mathrm n)\;=\;(\mathrm{TWA}\;(\mathrm n)\;/\;\mathrm{TWA}\;(0)\right)\;\times\;100$$

$$\mathrm{Wound}\;\mathrm{contraction}\;\mathrm{percentage}\;(\%\mathrm{WC})\;\mathrm{at}\;\mathrm{day}\;(\mathrm n)\:=\:100\;-\;\%\mathrm{WS}\;\left(\mathrm n\right)$$

$$\mathrm{Epithelialization}\;\mathrm{percentage}\;(\%\mathrm E)\;\mathrm{at}\;\mathrm{day}\;\left(\mathrm n\right)\;=\;(\mathrm{Epithelialization}\;\mathrm{area}\;\left(\mathrm n\right)\;/\;\mathrm{TWA}\;\left(\mathrm n\right))\;\times\;100$$

$$\mathrm{Non}-\mathrm{healing}\;\mathrm{percentage}\;\mathrm{at}\;\mathrm{day}\;\left(\mathrm n\right)\;=\;(\mathrm{Granulation}\;\mathrm{area}\;\left(\mathrm n\right)\;/\;\mathrm{TWA}\;(0))\;\times\;100$$

Healing percentage at day (n) = 100 - % of non-healing (n) [23]

Where: (n) refers to the specific assessment day, and (0) represents day 0.

All variables were measured at four time points: day 0, day 7, day 14, and day 21 following wound induction.

### Preparation of wound fluid

A standardized method for wound fluid collection was carried out at the clinical site, following a previously established protocol [7] on days 0, 5, 10, and 20. Briefly, the skin wounds were first cleansed with sterile water, after which an occlusive dressing was applied to the wound. After 10 min, the accumulated exudates beneath the dressing were collected by rinsing the wound with 1 mL of saline solution.

The collected wound fluid samples were then centrifuged at 14,000 × g for 10 min. The protein content of each sample was analyzed based on the method described by [24]. Aliquots were prepared and stored at -80 °C until further analysis. These wound fluid samples were subsequently used to assess total antioxidant capacity and lipid peroxidation markers.

### Assessment of oxidative stress biochemical markers in different groups

#### Assessment of total antioxidant capacity (TAC) (mM/L)

The total antioxidant capacity (TAC) was assessed through an enzymatic reaction in which the antioxidants present in the samples interacted with residual hydrogen peroxide (H₂O₂) in a colorimetric assay. This reaction led to the conversion of 3,5-dichloro-2-hydroxy benzenesulfonate into a colored product, the absorbance of which was measured at 505 nm using a UNICO-UV-2100 spectrophotometer, following the method described by [25].

#### Assessment of malondialdehyde (MDA) concentration (nM/ml)

Malondialdehyde (MDA) concentration was used as an index of lipid peroxidation as described by [26]. MDA was determined by measuring the thiobarbituric acid reactive species via (Bio Diagnostic kits, Giza, Egypt). The absorbance of the coloured product was measured at 505 nm by UNICO-UV-2100 spectrophotometer [25].

### Preparation of skin biopsies

Skin biopsies were prepared by rinsing the surface of each wound with normal saline before treatment began. Then biopsies were obtained at days 0, 5, 10, and 20 from the wounds in all groups under rigorous aseptic conditions. Samples were obtained using an 8-mm biopsy punch. Samples were taken from the wound margin, including the defect as well as 2–3 mm of normal skin, with a depth of 4 mm and stored at -80°c until gene expression analysis.

### Gene expression analysis

Quantitative real-time polymerase chain reaction (qPCR) for matrix extracellular phosphoglycoprotirn (MEPE), transforming growth factor-beta (TGF-β) and tumor necrosis factor-alpha (TNF-α):

Total RNA was extracted from skin biopsies using QIAmp RNA mini kit (QIAGEN, Hilden, Germany) as indicated by the manufacturer. Total RNA purity and concentration were obtained using a nanodrop ND-1000 spectrophotometer. The isolated RNA was used for cDNA synthesis using reverse transcriptase (Fermentas, EU). Real-time PCR (qPCR) was performed in a total volume of 20 µl using a mixture of 1 µl cDNA, 0.5 mM of each primer (Table 1), iQ SYBR Green Premix (Bio-Rad 170–880, USA). PCR amplification and analysis were achieved using Bio–Rad iCycler thermal cycler and the MyiQ realtime PCR detection system. Each assay included triplicate samples for each tested cDNAs and no-template negative control; the expression relative to control was calculated using the Eq. 2-ΔΔCT [27].

**Table 1:** Primer sequences of reference, Transforming growth factor-Beta (TGF-β), Tumor necrosis factor alpha (TNF-α) and matrix extracellular phosphoglycoprotein (MEPE) genes of Canis lupus familiaris

Target genes	Accession no.	Sequence (5’ to 3’)	Product size
GAPDH (Reference gene)	*XM_038448971.1*	**F: 5’- ATGGGCGTGAACCATGAGAA − 3’**	238 bp
		**R: 5’ CAGTGGAAGCAGGGATGATGT-3’**	
TNF-α	*NM_001003244.4*	**F: 5’ - GCCTCTTCTCCTTCCTCCTC − 3’**	159 bp
		**R: 5’ - TGTCACTTGGGGTTCGAGAA − 3’**	
TGF-β	*XM_038656896.1*	**F: 5’- TCAAGAAAAGTCCGCACAGC − 3’**	170 bp
		**R: 5’ - GCGCCAGGAATCATTGCTAT-3’**	
MEPE	*NM_001313825.1*	**F: 5’- TCTTTTCAGCGTGACTTGGGCA − 3’**	247 bp
		**R: 5’- AGGTGCTGGCTCTTGATTTCTTCT − 3’**	

### Measuring the level of canine platelets derived growth factor-bête (cPDGFβ)

Wound fluid PDGF levels were measured using a canine platelet growth factor subunit B, PDGFβ ELISA Kit (Catalog No: BZEK1862, Chongqing Biospes Co., Ltd, China) according to the manufacturer’s instructions. The optical density of the samples was recorded at a wavelength of 450 nm using a microplate reader (ELx800TM Absorbance Readers, BioTek Instruments, Inc., Vermont, USA). The sample concentration was calculated through the straight-line regression equation of the standard curve of the standard concentration and the OD value, with the sample OD value in the equation OD = a* Conc. + b where OD (Optical Density or Absorbance) is the measured absorbance of a sample at a specific wavelength, Conc. (Concentration): The concentration of the substance being measured, a (Slope): The proportionality constant that relates absorbance to concentration (determined from a calibration curve), b (Intercept): The absorbance value when the concentration is zero (background absorbance) [28].

### Sample preparation for histopathological evaluation of skin wounds

Skin wound tissue biopsy collected at 5, 10, and 20 days post-induction were fixed in 10% neutral buffered formalin and processed using the paraffin embedding technique. Tissue sections, 4 μm thick, were prepared using a rotary microtome and stained with hematoxylin and eosin (H&E) as well as Masson’s trichrome (MTC) stain.

For histological examination, a light microscope equipped with a digital camera was used. The positive area percentage of MTC-stained collagen was quantified using ImageJ software (*Developer: National Institutes of Health (NIH)*,* USA; Website*: https://imagej.nih.gov/ij/*).* Collagen measurement was performed on three captured images per wound at a 100× magnification power.

### Immunohistochemistry analysis

The expression of tumor necrosis factor-alpha (TNF-α) was assessed in wound tissues 5 days post-induction. Following antigen retrieval with citrate buffer (pH 6), anti-TNF-α antibody (1:100, Santa Cruz, USA) was applied to the deparaffinized tissue sections. Subsequently, the Universal Immuno-Detector DAB HRP Brown Detection System (*Bio SB*,* USA*) was used according to the manufacturer’s instructions.

The percentage of TNF-α positive area was analyzed using ImageJ software. Measurements were performed on three captured images per wound at a 200× magnification power.

### Statistical analysis

The data have been analyzed by the IBM SPSS Statistics 26 (IBM Corp, Armonk, NY, USA) program. The descriptive statistics were displayed in mean, and standard deviation for quantitative variables and percentage for the qualitative variables. Repeated measure ANOVA was used to find the significant difference in the effect of different protocols on the chemical and physical indices of wound healing including: Wound size percent, Wound contraction percent, wound healing percent and Epithelization percent, The chemical indices of wound healing including, Total antioxidant capacity (TAC) (mM/L), Malondialdhyde (MDA) concentration (nM/ml), Matrix extracellular phosphoglycoprotein (MEPE), Transforming growth factor beta (TGF-β), Tumor necrosis factor alpha (TNF-α) and Platelets –Derived Growth Factor Beta (PDGFβ) (pg/ml).

Furthermore, one-way ANOVA was used to find the significant difference between groups for each dependent variable, and if the results were significant Post Hoc Tukey test was used to find the result for between-group comparisons. All these dependent variables were measured four times, respectively, and the T test was used. The statistical significance was set at *p* < 0.05.

## Results

### Findings of SEM

**Fig. 1:**
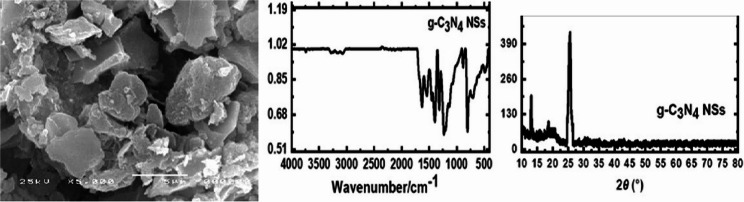
**a** SEM image of the prepared g-C3N4 NPs. **b** FTIR image of the prepared g-C3N4 NPs. **c** XRD image of the prepared g-C3N4 NPs

Graphitic carbon nitride (g-C3N4) exhibits a sheet-like structure, with stacked nanomaterial layers that enhance its surface area, making it ideal for nanoparticle doping. SEM imaging confirms its smooth, defect-free surface with minimal crevices (Fig .1a).

### Findings of FTIR

The FTIR spectrum of g-C3N4 shows several peaks at 810, 1145, 1213, 1393, 1587, and 1648 cm^− 1^. The peaks at 1145, 1213, 1393, 1587, and 1648 cm^− 1^ are associated with the stretching modes of CN heterocycles, which relate to the skeletal stretching vibrations of aromatic rings. In contrast, the peak at 810 cm^− 1^ corresponds to the breathing mode of the triazine units found in g-C3N4 nanomaterials (Fig. 1b).

### Findings of XRD

The X-ray diffraction (XRD) pattern of pure g-C3N4 nanomaterials reveals peaks at 26.73° and 13.37°. These peaks correspond to the (002) interlayer structural packing plane and the (100) interplanar stacking diffraction planes, respectively. The prominent peak at 26.73° indicates the stacking reflection of conjugated aromatic structures, suggesting a graphitic structure with an interlayer distance of 0.326 nm. (Fig. 1c).

### Results of PRP evaluation

The mean platelet count in PRP was 810 ± 45 × 10³/µL, while WBCs and RBCs were present in minimal amounts, measuring 30 ± 10/µL and 90 ± 20/µL, respectively.

### Clinical and planimetry findings

#### Clinical findings

**Fig. 2:**
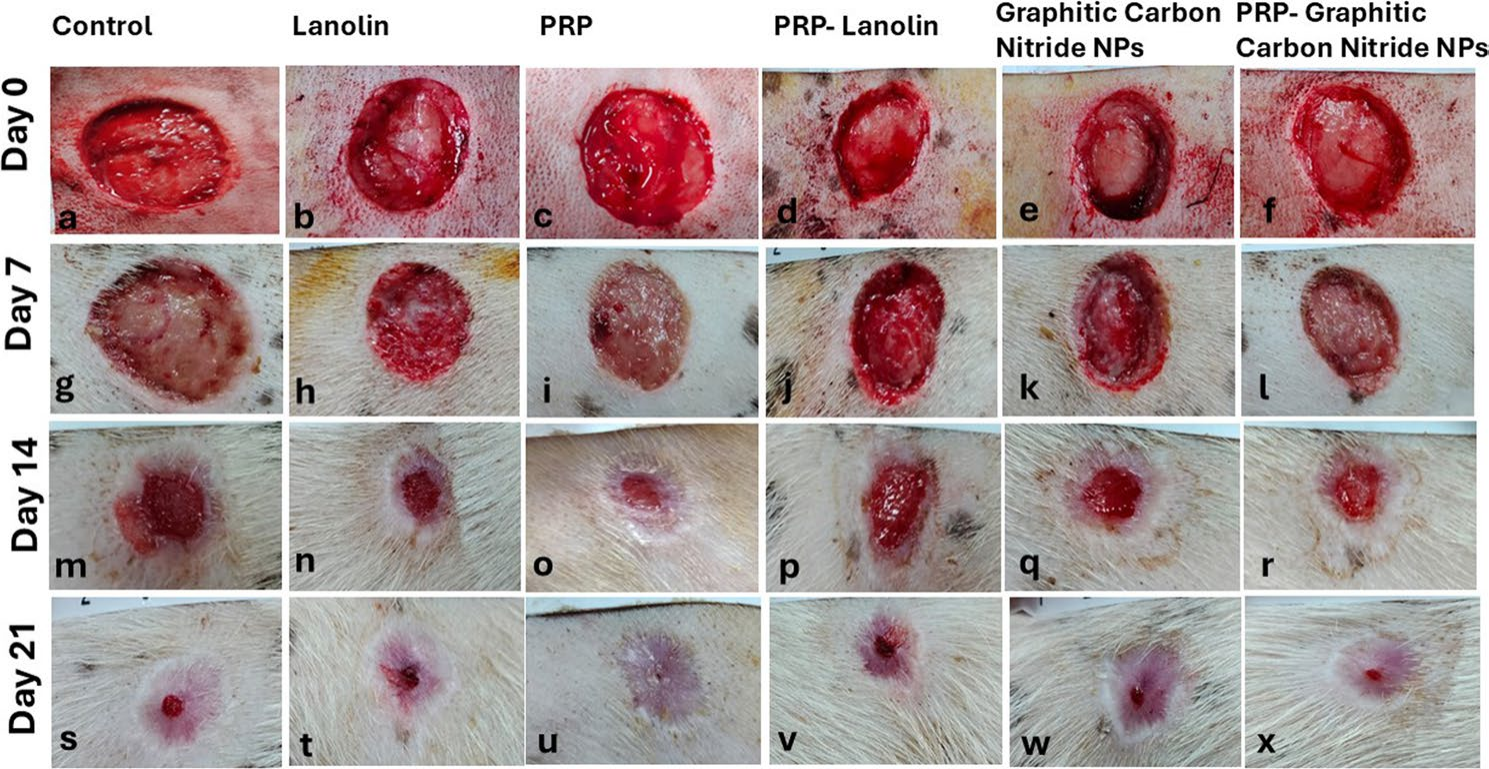
Representative photographs of wound healing in all groups at day 0 (**a-f**), 7 (**g-i**), 14 (**m-r**) and 21 (**s-x**)

On day 0, the wounds in all groups were of similar size (Fig. 2a, b, c, d, e, f). By days 7 and 14, groups 3 (PRP-treated), 5 (g-C3N4 NPs-treated), and 6 (PRP- g-C3N4 NPs treated) exhibited smaller wound sizes compared to group 1 (control), group 2 (lanolin-treated), and group 4 (PRP-lanolin treated). By day 21, complete wound healing and closure were observed in PRP-treated group, while g-C3N4 NPs-treated group, and PRP- g-C3N4 NPs treated group showed smaller wounds than the remaining groups (Fig. 2).

### Palnimetry findings

#### Wound size percent

There was a significant interaction effect of group and time for wound size percent (F = 5.437, *p* < 0.001). There was a significant main effect time for wound size percent (F = 4615.80, *p* < 0.001). At baseline (Day 0), all groups had identical wound sizes of 100.00 ± 0.00%, with no significant differences observed (P not computed).

By day 7, the PRP group exhibited the most pronounced reduction in wound size (68.18 ± 9.77), significantly lower than all other groups except PRP–g-C3N4 NPs (75.76 ± 1.79), (*P* ≤ 0.001). The g-C3N4 NPs (77.06 ± 2.24), PRP–lanolin (80.69 ± 5.23), control (83.39 ± 7.08), and lanolin (86.20 ± 4.17) groups showing large size wound with no significant difference between them (*P* > 0.05).

On day 14, PRP maintained its superiority with a wound size of (28.77 ± 2.31). PRP–g-C3N4 NPs (30.36 ± 2.40) and g-C3N4 NPs (31.43 ± 2.45%) showed statistically similar wound reduction, closely trailing PRP. PRP–lanolin exhibited moderate efficacy (35.32 ± 1.68), outperforming both lanolin (35.45 ± 4.45% and the control group (39.98 ± 3.21) but remaining significantly less effective than PRP and NP-containing groups, (*P* ≤ 0.001).

By day 21, PRP achieved the greatest overall wound closure with a final wound size of (23.30 ± 2.51), followed by PRP–g-C3N4 NPs (24.68 ± 0.80) and g-C3N4 NPs (25.70 ± 1.00) which were statistically different from other groups. PRP–lanolin showing moderate wound size reduction (28.34 ± 1.10) significantly different than lanolin (33.91 ± 1.47) and control (33.23 ± 0.78) which remained the least effective, with no significant difference between them, as shown in Table 2.

**Table 2:** Descriptive statistics for wound size %, wound contraction %, wound healing % and epithelialization % in all groups at days 0, 7, 14, and 21

Variables	Groups	Day 0	Day 7	Day 14	Day 21	*P*- Value
Wound size percent	Control	100 ^Aa^	83.385 ± 7.08^Bab^	39.977 ± 3.21 ^Ca^	33.227 ± 0.78 ^Da^	≤ 0.001 *
	Lanolin	100 ^Aa^	86.199 ± 4.17^Ba^	35.448 ± 4.45^Cab^	33.911 ± 1.47^Ca^	≤ 0.001 *
	PRP	100 ^Aa^	68.175 ± 9.77^Bc^	28.772 ± 2.31 ^Cd^	23.304 ± 2.51 ^Dc^	≤ 0.001 *
	PRP-lanolin	100^Aa^	80.69 ± 5.23^ab^	35.319 ± 1.68 ^Cabc^	28.335 ± 1.10 ^Db^	≤ 0.001 *
	g-C3N4 NPs	100^A a^	77.057 ± 2.24^Babc^	31.434 ± 2.45 ^Cbcd^	25.695 ± 1 ^Dc^	≤ 0.001 *
	PRP-g-C3N4 NPs	100 ^Aa^	75.76 ± 1.79^Bbc^	30.361 ± 2.40^Ccd^	24.678 ± 0.8 ^Cc^	≤ 0.001 *
	*P- Value*	Not computed	≤ 0.001 *	≤ 0.001 *	≤ 0.001 *	
Wound contraction percent	Control	0.00^Da^	16.615 ± 7.08^C bc^	60.023 ± 3.21^Bd^	66.773 ± 0.78 ^Ac^	≤ 0.001 *
	Lanolin	0.00^Ca^	13.801 ± 4.17 ^Bc^	64.552 ± 4.45^Acd^	66.089 ± 1.47 ^Ac^	≤ 0.001 *
	PRP	0.00^Da^	31.825 ± 9.77^Ca^	71.228 ± 2.31 ^Ba^	76.696 ± 2.51 ^Aa^	≤ 0.001 *
	PRP- lanolin	0.00^Da^	19.31 ± 5.23 ^Cbc^	64.681 ± 1.68 ^Bbcd^	71.665 ± 1.1^Ab^	≤ 0.001 *
	g-C3N4 NPs	0.00^Da^	22.943 ± 2.24^Cabc^	68.566 ± 2.45^Babc^	74.305 ± 1 ^Aa^	≤ 0.001 *
	PRP-g-C3N4 NPs	0.00^Da^	24.24 ± 1.79 ^Cab^	69.639 ± 2.40 ^Bab^	75.322 ± 0.8 ^Aa^	≤ 0.001 *
	*P*- value	Not computed	≤ 0.001 *	≤ 0.001 *	≤ 0.001 *	≤ 0.001 *
Healing percent	Control	0.00^Da^	23.24 ± 3.13 ^Cbc^	76.796 ± 0.27 ^Bc^	87.545 ± 3.65 ^Ac^	≤ 0.001 *
	Lanolin	0.00^Da^	18.601 ± 7.39 ^Cc^	80.158 ± 2.26 ^Bc^	87.546 ± 2.34 ^Ac^	≤ 0.001 *
	PRP	0.00^Da^	49.787 ± 10.55^C a^	92.976 ± 1.47^B a^	99.509 ± 0.39 ^Aa^	≤ 0.001 *
	PRP-lanolin	0.00^Da^	27.739 ± 8.81^C bc^	86.1 ± 1.75^B b^	92.679 ± 2.6 ^Ab^	≤ 0.001 *
	g-C3N4 NPs	0.00^Da^	28.257 ± 2.01^C bc^	87.888 ± 2.93^B b^	96.662 ± 2.12^Aab^	≤ 0.001 *
	PRP-g-C3N4 NPs	0.00^Da^	32.827 ± 2.44 ^Cb^	89.582 ± 1.33^B ab^	97.462 ± 0.94 ^Aa^	≤ 0.001 *
	*P*- value	Not computed	≤ 0.001 *	0.001 *	≤ 0.001 *	
Epithelialization percent	Control	0.00^Da^	8.357 ± 3.74 ^C b^	16.773 ± 1.52 ^B b^	20.771 ± 3.17^A a^	≤ 0.001 *
	Lanolin	0.00^Da^	4.800 ± 3.93 ^C b^	15.606 ± 2.98 ^B b^	21.457 ± 2.24^A a^	≤ 0.001 *
	PRP	0.00^Da^	17.312 ± 1.06 ^C a^	21.747 ± 2.32 ^B a^	22.814 ± 2.26 ^Aa^	≤ 0.001 *
	PRP-lanolin	0.00 ^Ca^	8.429 ± 4.31 ^B b^	21.419 ± 1.59 ^Aa^	21.014 ± 2.40 ^Aa^	≤ 0.001 *
	g-C3N4 NPs	0.00 ^Ca^	5.314 ± 0.57 ^B b^	19.322 ± 3.58 ^Aab^	22.357 ± 2.06^A a^	≤ 0.001 *
	PRP-g-C3N4 NPs	0.00^Da^	8.587 ± 3.05 ^C b^	19.943 ± 2.57 ^B ab^	22.14 ± 1.57^A a^	≤ 0.001 *
	*P*- value	Not computed	≤ 0.001 *	≤ 0.001 *	0.618	

Means with different capital letters in the same row indicate significant difference

Means with different small letters in the same column indicate significant difference

*Significant at *P* < 0.05

Throughout the study, all changes over time within groups and all intergroup differences (except at Day 0) were statistically significant (*P* ≤ 0.001), confirming the superior wound-reducing effect of PRP and the supportive role of g-C3N4 nanoparticles in accelerating healing.

#### Wound contraction percent

There was a significant interaction effect of group and time for Wound contraction percent (F = 5.437, *p* < 0.001). There was a significant main effect time for Wound contraction percent (F = 4615.8, *p* < 0.001). All groups began at baseline (Day 0) with 0.00 ± 0.00% wound contraction and showed no differences between treatments (P not computed).

By day 7 (*P* ≤ 0.001), the PRP group exhibited the most rapid early wound contraction (31.83 ± 9.77), significantly outperforming all other groups except PRP–g-C3N4 NPs (24.24 ± 1.79%), which showed better performance than g-C3N4 NPs (22.94 ± 2.24) (*P* ≤ 0.001). The PRP–lanolin group (19.31 ± 5.23) following them and not significantly different from the control group (16.62 ± 7.08) and lanolin-treated wounds which showed the lowest contraction at this stage (13.80 ± 4.17), (*P* > 0.05).

On day 14, PRP continued to lead with (71.23 ± 2.31) contraction and significantly different from all except PRP–g-C3N4 NPs (69.64 ± 2.40) and g-C3N4 NPs (68.57 ± 2.45) (*P* ≤ 0.001). The PRP–lanolin group (64.68 ± 1.68) followed closely and not significantly different from all groups (*P* > 0.05). The control (60.02 ± 3.21) and lanolin (64.55 ± 4.45) had a similar contraction value and remained statistically inferior to PRP.

By day 21, PRP maintained its lead with (76.70 ± 2.51) contraction. PRP–g-C3N4 NPs (75.32 ± 0.80%) and g-C3N4 NPs (74.31 ± 1.00) reached comparable final values to PRP (*P* > 0.05). PRP–lanolin achieved (71.67 ± 1.10) significantly better than both the control (66.77 ± 0.78) and lanolin (66.09 ± 1.47) (*P* ≤ 0.001), as shown in Table 2.

In summary, PRP alone induced the fastest and most substantial wound contraction, particularly evident by day 7 and persisting through day 21. PRP–g-C3N4 NPs and g-C3N4 NPs also achieved excellent final outcomes, statistically similar to PRP. PRP–lanolin offered moderate benefit, superior to Lanolin alone but not matching PRP efficacy. Lanolin and control showed the lowest contraction rates throughout the study period.

#### Healing percent

There was a significant interaction effect of group and time for healing percent (F = 11.945, *p* < 0.001). There was a significant main effect time for healing percent (F = 6280.21, *p* < 0.001). At baseline (day 0), all groups started at 0.00 ± 0.00% healing, with no significant differences among them (P not computed).

By day 7, the PRP group demonstrated the highest healing percentage at (49.79 ± 10.55), significantly outperforming all other treatments, (*P* ≤ 0.001). PRP–g-C3N4 NPs followed with (32.83 ± 2.44), showing better healing than g-C3N4 NPs (28.26 ± 2.01), PRP–lanolin (27.74 ± 8.81), control (23.24 ± 3.13), and lanolin (18.60 ± 7.39), the latter being the least effective at this stage and significantly different than PRP and PRP–g-C3N4 NPs treated groups, (*P* ≤ 0.001). .

By day 14, PRP remained superior with a healing percentage of (92.98 ± 1.47), significantly better than all groups except PRP–g-C3N4 NPs (89.58 ± 1.33) (*P* = 0.001). g-C3N4 NPs also showed robust healing (87.89 ± 2.93) followed with PRP-lanolin (86.1 ± 1.75) which were statistically different than lanolin (80.16 ± 2.26%) and control (76.80 ± 0.27%) (*P* ≤ 0.001).

By day 21, PRP reached nearly complete healing at (99.51 ± 0.39), significantly higher than all other groups except PRP–g-C3N4 NPs (97.46 ± 0.94) and g-C3N4 NPs (96.66 ± 2.12), which achieved similarly high final values%) (*P* ≤ 0.001). PRP–lanolin (92.68 ± 2.60) showed moderate improvement but remained below PRP and NP groups but significantly higher percentage more than control (87.55 ± 3.65) and lanolin (87.55 ± 2.34) groups, that recorded the lowest final healing percentages, with no significant difference between them, as shown in Table 2.

Overall, PRP treatment was the most effective, demonstrating a fast and consistent increase in healing across all timepoints. Treatments involving g-C3N4 NPs, whether alone or in combination with PRP, showed strong performance and closely approached PRP’s efficacy by the end.

#### Epithelialization percent

There was a significant interaction effect of group and time for epithelization percent (F = 6.343, *p* < 0.008). There was a significant main effect time for epithelization percent (F = 744.665, *p* < 0.001). At day 0, all groups showed 0.00% epithelialization with no significant differences (P not computed).

By Day 7, the PRP group achieved the highest early epithelialization percentage (17.31 ± 1.06), significantly surpassing all other groups (*P* ≤ 0.001). PRP–g-C3N4 NPs (8.59 ± 3.05), PRP–lanolin (8.43 ± 4.31), and the control (8.36 ± 3.74%) showed similar moderate responses, while g-C3N4 NPs (5.31 ± 0.57) and lanolin (4.80 ± 3.93) demonstrated the lowest epithelialization values, and all not significantly different from each other (*P* > 0.05).

By day 14, PRP maintained its lead with (21.75 ± 2.32) followed by PRP–lanolin (21.42 ± 1.59), which were significantly higher than control (16.77 ± 1.52) and lanolin (15.61 ± 2.98) (*P* ≤ 0.001). Intermediate epithelialization levels were observed in, PRP–g-C3N4 NPs (19.94 ± 2.57), and g-C3N4 NPs (19.32 ± 3.58) with no significant difference between them and all groups (*P* > 0.05).

However, by day 21 (*P* = 0.618), no significant differences were observed between groups (*P* = 0.618), as all reached similar epithelialization levels. Final values ranged from 22.81 ± 2.26 in the PRP group to 20.77 ± 3.17 in the control, with all other groups falling in between.

This indicates that while PRP treatment significantly accelerated early epithelialization, all groups ultimately achieved comparable epithelial coverage by the end of the study. Treatments involving g-C3N4 NPs, whether alone or in combination, showed progressive improvement and nearly matched PRP by day 21 as shown in Table 2.

### Biochemical markers

#### Total Antioxidant Capacity (TAC)

There was a significant interaction effect of group and time for TAC (F = 20.80, *p* < 0.001). There was a significant main effect time for TAC (F = 171.137, *p* < 0.001). At day 0, there were no statistically significant differences in TAC values among the groups (*P* = 0.082), with all starting from relatively similar levels: Control (1.16 ± 0.31), lanolin (1.47 ± 0.16), PRP (1.52 ± 0.18), PRP–lanolin (1.67 ± 0.45), g-C3N4 NPs (1.79 ± 0.12), and PRP–g-C3N4 NPs (1.73 ± 0.30).

By day 5, the differences became highly significant. The control group exhibited the highest TAC (1.89 ± 0.11), significantly greater than all others (*P* < 0.001), followed by lanolin (1.59 ± 0.07), which was significantly higher than all the rest groups. Nano formulations demonstrated intermediate TAC values: PRP–g-C3N4 NPs (0.90 ± 0.08), and g-C3N4 NPs (0.78 ± 0.15), also PRP–lanolin demonstrated intermediate TAC values (0.70 ± 0.15), all significantly higher than PRP but significantly lower than control and lanolin. PRP showed a marked reduction in TAC to 0.32 ± 0.02, indicating substantial antioxidant consumption and elevated oxidative stress (*P* < 0.001 vs. all groups).

On day 10, lanolin maintained the highest TAC (1.34 ± 0.08) significantly greater than other treatments (*P* ≤ 0.001). The nano formulations, including g-C3N4 NPs (0.62 ± 0.18), and PRP–g-C3N4 NPs (0.58 ± 0.26), and also PRP–lanolin (0.65 ± 0.15), maintained higher TAC levels than PRP, with no significant differences were found among them, (*P* > 0.05). Following them, the control (0.07 ± 0.006) and PRP group reached its lowest TAC level (0.01 ± 0.01), remaining significantly below all groups.

By day 20, control became again the highest TAC (1.26 ± 0.06) and was significantly higher than all other groups (*P* ≤ 0.001). following that the lanolin group (0.37 ± 0.10) maintained high TAC with significant difference with control and PRP group which remained lowest TAC (0.20 ± 0.017). Nano formulations again performed moderate TAC with PRP–g-C3N4 NPs (0.28 ± 0.03), and g-C3N4 NPs (0.23 ± 0.05) and also PRP–lanolin (0.29 ± 0.02), showing similar values (*P* > 0.05), all significantly greater than PRP but lower than control (*P* ≤ 0.001), as shown in Table 3.

**Table 3:** Descriptive statistics for biochemical factors in all groups at days 0, 5, 10, and 20

	Groups	Day 0	Day 5	Day 10	Day 20	*P- value*
TAC (mM/L)	CT	1.16 ± 0.31^ABa^	1.89 ± 0.11^Aa^	0.07 ± 0.006 ^Cc^	1.26 ± 0.06 ^Ba^	≤0.001*
	Lanolin	1.47 ± 0.16^ABa^	1.59 ± 0.07^Ab^	1.34 ± 0.08 ^Ba^	0.37 ± 0.10 ^Cb^	≤ 0.001*
	PRP	1.52 ± 0.18^Aa^	0.32 ± 0.02 ^Bd^	0.01 ± 0.01^Dc^	0.20 ± 0.017 ^Cc^	≤ 0.001*
	PRP-lanolin	1.67 ± 0.45^Aa^	0.70 ± 0.15^Ac^	0.65 ± 0.15^ABb^	0.29 ± 0.02 ^Bbc^	≤ 0.001*
	g-C3N4 NPs	1.79 ± 0.12^Aa^	0.78 ± 0.15 ^Bc^	0.62 ± 0.18 ^Bb^	0.23 ± 0.05 ^Cbc^	≤ 0.001*
	PRP-g-C3N4 NPs	1.73 ± 0.30^Aa^	0.90 ± 0.08 ^Bc^	0.58 ± 0.26 ^BCb^	0.28 ± 0.03 ^Cbc^	0.001*
*P*- value		0.082	≤ 0.001 *	≤ 0.001 *	≤ 0.001 *	
MDA (nM/ml)	CT	31.56 ± 1.93 ^Aba^	37.22 ± 1.69 ^Aa^	5.37 ± 0.26^Cb^	2.17 ± 0.10 ^Bb^	≤ 0.001 *
	Lanolin	31.90 ± 0.13^A Ba^	18.90 ± 2.8 ^Abc^	11.04 ± 1.61 ^Ba^	5.31 ± 0.86^Ca^	≤ 0.001 *
	PRP	31.80 ± 4.68 ^Aa^	14.67 ± 1.26 ^Bc^	2.05 ± 0.15^Dcd^	0.74 ± 0.06^Cc^	≤ 0.001 *
	PRP-lanolin	31.86 ± 1.38 ^Aa^	20.20 ± 1.91 ^Ab^	3.63 ± 0.52 ^A Bbc^	0.22 ± 0.04 ^Bc^	≤ 0.001 *
	g-C3N4 NPs	1 ^Aa^	1.37 ± 0.21 ^Bd^	1.78 ± 0.50 ^Bd^	2.45 ± 1.1^Cc^	≤ 0.001 *
	PRP-g-C3N4 NPs	27.74 ± 1.77 ^Aa^	5.17 ± 0.91 ^Bd^	1.23 ± 0.11 ^BCd^	0.06 ± 0.05^Cc^	≤ 0.001 *
*P*- value		0.398	≤ 0.001 *	≤ 0.001 *	≤ 0.001 *	
MEPE	CT	1 ^Ba^	1.49 ± 0.04 ^Ab^	0.31 ± 0.02 ^Cc^	0.14 ± 0.02^Dc^	≤ 0.001 *
	Lanolin	1 ^A Ba^	1.45 ± 0.20 ^Ab^	0.78 ± 0.18 ^Bbc^	0.11 ± 0.01 ^Cc^	≤ 0.001 *
	PRP	1 ^Ca^	13.43 ± 1.75 ^Aa^	1.50 ± 0.58 ^Cab^	4.19 ± 0.3 ^Ba^	≤ 0.001 *
	PRP-lanolin	1 ^CDa^	1.63 ± 0.17 ^A Bb^	1.49 ± 0.03 ^Aab^	1.15 ± 0.14 ^BCbc^	≤ 0.001 *
	g-C3N4 NPs	1 ^Aa^	1.37 ± 0.21 ^Ab^	1.78 ± 0.50 ^Aab^	2.45 ± 1.1 ^Aab^	≤ 0.001 *
	PRP-g-C3N4 NPs	1 ^Ba^	3.23 ± 0.25 ^Ab^	2.28 ± 0.66 ^A Ba^	2.72 ± 1.5 ^ABab^	≤ 0.001 *
*P*- value		Not computed	≤ 0.001 *	≤ 0.001 *	≤ 0.001 *	
TGF-β	CT	1 ^Ba^	1.85 ± 0.02 ^Abc^	0.49 ± 0.16^Cd^	0.30 ± 0.17 ^Dd^	≤ 0.001 *
	Lanolin	1 ^Aa^	0.08 ± 0.01^Cc^	0.09 ± 0.009 ^Be^	0.16 ± 0.06 ^BCd^	≤ 0.001 *
	PRP	1 ^Da^	8.47 ± 0.13 ^Ba^	9.60 ± 0.20 ^Aa^	7.11 ± 0.29^Ca^	≤ 0.001 *
	PRP-lanolin	1^Ca^	1.24 ± 0.07 ^Bc^	1.54 ± 0.02 ^Ac^	0.10 ± 0.01 ^Dd^	≤ 0.001 *
	g-C3N4 NPs	1^Da^	2.26 ± 0.17 ^Abc^	0.79 ± 0.02^Cd^	0.91 ± 0.01 ^Bc^	≤ 0.001 *
	PRP-g-C3N4 NPs	1 ^Aa^	3.99 ± 2.29 ^ABCb^	5.10 ± 0.39^Bb^	2.74 ± 0.13 ^Cb^	≤ 0.001 *
*P*- value		Not computed	≤ 0.001 *	≤ 0.001 *	≤ 0.001 *	
TNF-α	CT	1 ^Ca^	0.47 ± 0.06 ^Dcd^	18.09 ± 0.18 ^Aa^	6.14 ± 0.22 ^Ba^	≤ 0.001 *
	Lanolin	1 ^Da^	1.51 ± 0.05 ^Cb^	1.78 ± 0.04 ^Bb^	2.33 ± 0.30 ^Ab^	≤ 0.001 *
	PRP	1 ^Aa^	0.57 ± 0.03 ^Bcd^	0.58 ± 0.07 ^Bc^	0.11 ± 0.01 ^Cc^	≤ 0.001 *
	PRP-lanolin	1 ^Aa^	0.98 ± 0.05 ^Abc^	0.09 ± 0.01 ^Be^	0.23 ± 0.02 ^Cc^	≤ 0.001 *
	g-C3N4 NPs	1 ^Ba^	4.98 ± 0.60 ^Aa^	0.34 ± 0.04 ^Cd^	0.47 ± 0.15 ^Cc^	≤ 0.001 *
	PRP-g-C3N4 NPs	1 ^Aa^	0.24 ± 0.15 ^Bd^	0.18 ± 0.04 ^Bde^	0.19 ± 0.02 ^Bc^	≤ 0.001 *
*P*- value		Not computed	≤ 0.001 *	≤ 0.001 *	≤ 0.001 *	
PDGFβ	CT	12.03 ± 1.42 ^Ca^	35.77 ± 3.01 ^Ab^	26.03 ± 2.7 ^Bc^	12.27 ± 1.62 ^Cc^	≤ 0.001 *
	Lanolin	12.00 ± 4.36 ^Ca^	33.07 ± 1.29 ^Ab^	27.97 ± 1.18 ^Bc^	13.97 ± 1 ^Cc^	≤ 0.001 *
	PRP	12.70 ± 5.88 ^Da^	81.83 ± 5 ^Ca^	130.33 ± 5.68 ^Ba^	169.1 ± 8.35 ^Aa^	≤ 0.001 *
	PRP-lanolin	13.53 ± 3.14 ^Da^	71.33 ± 4.04 ^Ca^	104.33 ± 7.50 ^Bb^	132.17 ± 10.54 ^Ab^	≤ 0.001 *
	g-C3N4 NPs	13.33 ± 4.72 ^Ba^	37.67 ± 2.52 ^Ab^	32 ± 3 ^Ac^	14.27 ± 1.10 ^Bc^	≤ 0.001 *
	PRP-g-C3N4 NPs	11.80 ± 2.43 ^Da^	82 ± 6 ^Ca^	118.33 ± 4.72 ^Ba^	163.6 ± 9.91 ^Aa^	≤ 0.001 *
*P*- value		0.993	≤ 0.001 *	≤ 0.001 *	≤ 0.001 *	

Means with different capital letters in the same row indicate a significant difference. Means with different small letters in the same column indicate a significant difference

*Significant at *P* < 0.05

In conclusion, PRP alone resulted in the lowest TAC across all post-injury timepoints, reflecting intense oxidative burden and antioxidant depletion. In contrast, nano formulations, especially PRP–g-C3N4 NPs, preserved significantly higher TAC levels, indicating improved oxidative balance. Control and lanolin retained higher TAC values overall, but this likely reflects limited metabolic stress rather than active tissue remodeling. The best redox balance was achieved with PRP–g-C3N4 NPs, which successfully limited TAC depletion while supporting an effective wound healing responseas.

#### Malondialdehyde (MDA)

There was a significant interaction effect of group and time for MDA (F = 53.63, *p* < 0.001). There was a significant main effect time for MDA (F = 1730.91, *p* < 0.001). At day 0, there were no statistically significant differences in malondialdehyde (MDA) levels among the groups (*P* = 0.398), with values ranging from lanolin (31.90 ± 0.13), PRP–lanolin (31.86 ± 1.38), PRP (31.80 ± 4.68), control (31.56 ± 1.93), g-C3N4 NPs (31.00 ± 1.00), to PRP–g-C3N4 NPs (27.74 ± 1.77).

By day 5, significant differences emerged, with the control group displaying the highest MDA level (37.22 ± 1.69) significantly higher than all groups, indicating the greatest oxidative stress. Following that PRP–lanolin (20.20 ± 1.91) which was significantly higher than all groups except lanolin group (18.90 ± 2.80) that showed an elevated MDA with no significant difference with PRP (14.67 ± 1.26) (*P* > 0.05). The most substantial reduction was seen in PRP–g-C3N4 NPs (5.17 ± 0.91) and g-C3N4 NPs (1.37 ± 0.21), both significantly lower than all other groups (*P* ≤ 0.001), demonstrating strong lipid peroxidation suppression.

On day 10, lanolin (11.04 ± 1.61) was significantly higher than all other treatments (*P* ≤ 0.001). Following that the control group (5.37 ± 0.26) which also significantly different from all other treatments. PRP–lanolin (3.63 ± 0.52) showed higher MDA than the PRP (2.05 ± 0.15) both not significant different from each other (*P* > 0.05), but significantly different from both g-C3N4 NPs (1.78 ± 0.50) and PRP–g-C3N4 NPs (1.23 ± 0.11) (*P* ≤ 0.001).

By day 20, Lanolin (5.31 ± 0.86) continued to be the highest MDA value than other groups (*P* ≤ 0.001). Control (2.17 ± 0.10) followed lanolin and significantly different from other groups. Following that PRP (0.74 ± 0.06), g-C3N4 NPs (0.48 ± 0.03), PRP–lanolin (0.22 ± 0.04) and PRP–g-C3N4 NPs achieved the most profound suppression of MDA (0.06 ± 0.05), all are not significant from each other (*P* > 0.05), as shown in Table 3.

In conclusion, PRP–g-C3N4 NPs consistently maintained the lowest MDA levels across all post-baseline timepoints, indicating the most effective oxidative stress control. PRP and g-C3N4 NPs also showed strong antioxidant effects, markedly reducing MDA from day 5 to day 20. PRP–lanolin demonstrated moderate antioxidant potential, consistently better than lanolin alone, but less effective than nano formulations. Lanolin and control groups exhibited the highest oxidative damage, with persistently elevated MDA throughout the study (*P* ≤ 0.001).

### Gene expression

#### Matrix Extracellular Phosphoglycoprotein (MEPE)

There was a significant interaction effect of group and time for MEPE (F = 58.60, *p* < 0.001). There was a significant main effect time for MEPE (F = 102.21, *p* < 0.001). At day 0, all groups started with equal baseline MEPE expression levels (1.00), and no significant differences were observed, as expected (*P* > 0.05).

By day 5, significant up-regulation of MEPE occurred, with PRP showing the highest expression (13.43 ± 1.75), significantly greater than all other groups (*P* < 0.001). PRP–g-C3N4 NPs followed with elevated expression (3.23 ± 0.25), markedly higher than control and lanolin, but much lower than PRP. All other groups had low MEPE expression: PRP–lanolin (1.63 ± 0.17), control (1.49 ± 0.04), lanolin (1.45 ± 0.20), and g-C3N4 NPs (1.37 ± 0.21), with no significant differences among them (*P* > 0.05).

By day 10, PRP–g-C3N4 NPs exhibited the highest MEPE level (2.28 ± 0.66), significantly different with control (0.31 ± 0.02) lowest and lanolin (0.78 ± 0.18) which remained the lowest (*P* ≤ 0.001). g-C3N4 NPs (1.78 ± 0.50), PRP (1.50 ± 0.58), PRP–lanolin (1.49 ± 0.03) and even lanolin showed similar expression levels without significant differences among them (*P* > 0.05).

By day 20, PRP again recorded the strongest MEPE expression (4.19 ± 0.30), significantly higher than all groups except PRP–g-C3N4 NPs (2.72 ± 1.50) and g-C3N4 NPs (2.45 ± 1.10) (*P* < 0.001). PRP–lanolin showed lower expression (1.15 ± 0.14), still significantly lower than only PRP. Lanolin (0.11 ± 0.01) and control (0.14 ± 0.02) exhibit the lowest MEPE expression, with no statistical difference between them and also PRP–lanolin (*P* > 0.05), but. significantly different from the rest of treated groups (*P* ≤ 0.001), as shown in Table 3.

In conclusion, PRP alone induced the most potent and sustained MEPE upregulation, particularly evident at days 5 and 20. PRP–g-C3N4 NPs also significantly enhanced MEPE expression, peaking on day 10 and maintaining strong levels by day 20. g-C3N4 NPs matched PRP–g-C3N4 NPs at later timepoints, though their early induction was weaker. PRP–lanolin showed moderate expression, while lanolin and control demonstrated minimal MEPE activity, suggesting limited activation of matrix-related genes.

#### Transforming Growth Factor Beta (TGF-β)

There was a significant interaction effect of group and time for TGF-β (F = 35.72, *p* < 0.001). There was a significant main effect time for TGF-β (F = 68.96, *p* < 0.001). At day 0, all groups began with normalized TGF-β expression values of 1.00, with no statistically significant differences observed (*P* > 0.05).

By day 5, highly significant differences emerged. PRP showed the strongest induction of TGF-β (8.47 ± 0.13), significantly higher than all other groups (*P* < 0.001). PRP–g-C3N4 NPs followed with moderately high expression (3.99 ± 2.29), significantly lower than PRP but higher than the rest of the treatments. g-C3N4 NPs (2.26 ± 0.17) and the control group (1.85 ± 0.02) showed moderate TGF-β levels, while PRP–lanolin (1.24 ± 0.07) and lanolin (0.08 ± 0.01) had the lowest TGF-β expression (0.08 ± 0.01), significantly below PRP and PRP–g-C3N4 NPs groups.

By day 10, all groups were significantly different from each other except g-C3N4 NPs and control (*P* ≤ 0.001). PRP maintained the highest TGF-β expression (9.60 ± 0.20), clearly outperforming all others. PRP–g-C3N4 NPs (5.10 ± 0.39) showed a strong but lower expression, while PRP–lanolin (1.54 ± 0.02) and g-C3N4 NPs (0.79 ± 0.02) exhibited moderate to low expression. Control (0.49 ± 0.16) and lanolin (0.09 ± 0.009) remained the lowest, indicating poor regenerative signaling.

By day 20 (*P* ≤ 0.001), statistically significant differences persisted. PRP (7.11 ± 0.29) continued to demonstrate the highest TGF-β levels, indicating sustained regenerative signaling. PRP–g-C3N4 NPs (2.74 ± 0.13) remained the second most effective, significantly higher than g-C3N4 NPs (0.91 ± 0.01), which showed moderate TGF-β induction. Surprisingly, PRP–lanolin expression dropped sharply by this stage (0.10 ± 0.01), similar to lanolin (0.16 ± 0.06) and control (0.30 ± 0.17) with no significant difference between them, as shown in Table 3.

In conclusion, PRP treatment resulted in the most robust and sustained TGF-β expression across all timepoints, highlighting its strong regenerative signaling (*P* ≤ 0.001). PRP–g-C3N4 NPs provided the second-best performance, maintaining effective expression up to day 20. g-C3N4 NPs had moderate and earlier peaking induction. PRP–lanolin showed an early rise in TGF-β but experienced a sharp decline by day 20. Lanolin and control groups demonstrated minimal TGF-β activity, suggesting poor healing response.

#### Tumor Necrosis Factor Alpha (TNF α)

There was a significant interaction effect of group and time for TNF-α (F = 1530.11, *p* < 0.001). There was a significant main effect time for TNF-α (F = 865.81, *p* < 0.001). On day 0, all groups had equal TNF-αexpression levels (1.00), as expected due to normalization, with no statistical differences between them (*P* > 0.05).

By day 5, g-C3N4 NPs showed the highest TNF-α expression (4.98 ± 0.60), indicating strong inflammatory activity, significantly higher than all other groups (*P* < 0.001). Lanolin followed with increased TNF-α levels (1.51 ± 0.05), also higher than all except PRP–lanolin which showed moderate TNF-α (0.98 ± 0.05), higher than control (0.47 ± 0.06), PRP (0.57 ± 0.03) with no significant difference between them (*P* > 0.05). PRP–g-C3N4 NPs (0.24 ± 0.15 significantly different with g-C3N4 NPs and lanolin groups demonstrated the lowest inflammatory markers, indicating early suppression.

By day 10, all groups are statistically significant from each other except between PRP–g-C3N4 NPs, g-C3N4 NPs and PRP–lanolin (*P* ≤ 0.001). The control group had the highest TNF-α expression (18.09 ± 0.18), reflecting unresolved inflammation. Lanolin (1.78 ± 0.04) remained elevated, while PRP (0.58 ± 0.07) and PRP–g-C3N4 NPs (0.18 ± 0.04) showed substantial suppression. g-C3N4 NPs (0.34 ± 0.04) showed a moderate reduction, better than lanolin and control but still higher than PRP-based treatments. PRP–lanolin exhibited the lowest TNF-α level overall (0.09 ± 0.01).

By day 20. The control group (6.14 ± 0.22) and lanolin (2.33 ± 0.30) continued to exhibit high TNF-α expression, indicating chronic inflammation and there were statistically significant difference between them and all the rest groups (*P* ≤ 0.001). g-C3N4 NPs (0.47 ± 0.15) remained effective in reducing TNF-α but were slightly less potent than PRP treatments. following that PRP–lanolin (0.23 ± 0.02), PRP–g-C3N4 NPs (0.19 ± 0.02) and PRP (0.11 ± 0.01), which had the lowest TNF-α levels, with no significant difference among them (*P* > 0.05). as illustrated in Table 3.

In conclusion, PRP–lanolin demonstrated the fastest and most consistent TNF-α suppression, especially evident by day 10. PRP and PRP–g-C3N4 NPs also provided excellent control of inflammatory response, particularly by day 20. g-C3N4 NPs showed delayed anti-inflammatory action, while lanolin and control consistently exhibited the highest TNF-α expression across all timepoints, reflecting sustained inflammation.

### Platelets Derived Growth Factor Bête (PDGFβ)

There was a significant interaction effect of group and time for PDGFβ (F = 242.09, *p* < 0.001). A significant main effect of time was also observed for PDGFβ (F = 1264.29, *p* < 0.001). At day 0, there were no statistically significant differences among groups in platelet-derived growth factor beta (PDGFβ) levels (*P* = 0.993). All groups exhibited similar baseline values: Control (12.03 ± 1.42), lanolin (12.00 ± 4.36), PRP (12.70 ± 5.88), PRP–lanolin (13.53 ± 3.14), g-C3N4 NPs (13.33 ± 4.72), and PRP–g-C3N4 NPs (11.80 ± 2.43).

By day 5, group differences became highly significant. PRP–g-C3N4 NPs (82 ± 6), PRP (81.83 ± 5.00) and PRP–lanolin (71.33 ± 4.04) induced the highest PDGFβ levels, with no statistical difference between them (*P* < 0.05). Also, Moderate induction was observed in g-C3N4 NPs (37.67 ± 2.52), lanolin (33.07 ± 1.29) and the control (35.77 ± 3.01) with no significant difference between them, but all were significantly different from other groups (*P* ≤ 0.001).

By day 10, the trend persisted, with PRP (130.33 ± 5.68) and PRP–g-C3N4 NPs (118.33 ± 4.72) maintaining the highest PDGFβ levels, statistically comparable to each other. PRP–lanolin (104.33 ± 7.50) followed closely but was significantly lower than PRP, (*P* ≤ 0.001). g-C3N4 NPs (32.00 ± 3.00), lanolin (27.97 ± 1.18), and control (26.03 ± 2.70) remained substantially lower and all were significantly different from other groups (*P* ≤ 0.001).

By day 20, PRP (169.1 ± 8.35) and PRP–g-C3N4 NPs (163.6 ± 9.91) continued to show the strongest PDGFβ expression, with no significant difference between them. PRP–lanolin (132.17 ± 10.54) was notably effective but remained significantly lower than PRP (*P* < 0.001). g-C3N4 NPs (14.27 ± 1.10), lanolin (13.97 ± 1.00), and control (12.27 ± 1.62) showed minimal PDGFβ expression, with no significant differences among them, (*P* > 0.05), as shown in Table 3.

Overall, the results highlight the potent and sustained PDGFβ inducing capability of PRP, even when combined with g-C3N4 NPs, while PRP–lanolin offered moderate induction, and other treatments showed minimal effects throughout the study.

### Histopathological findings

**Fig. 3:**
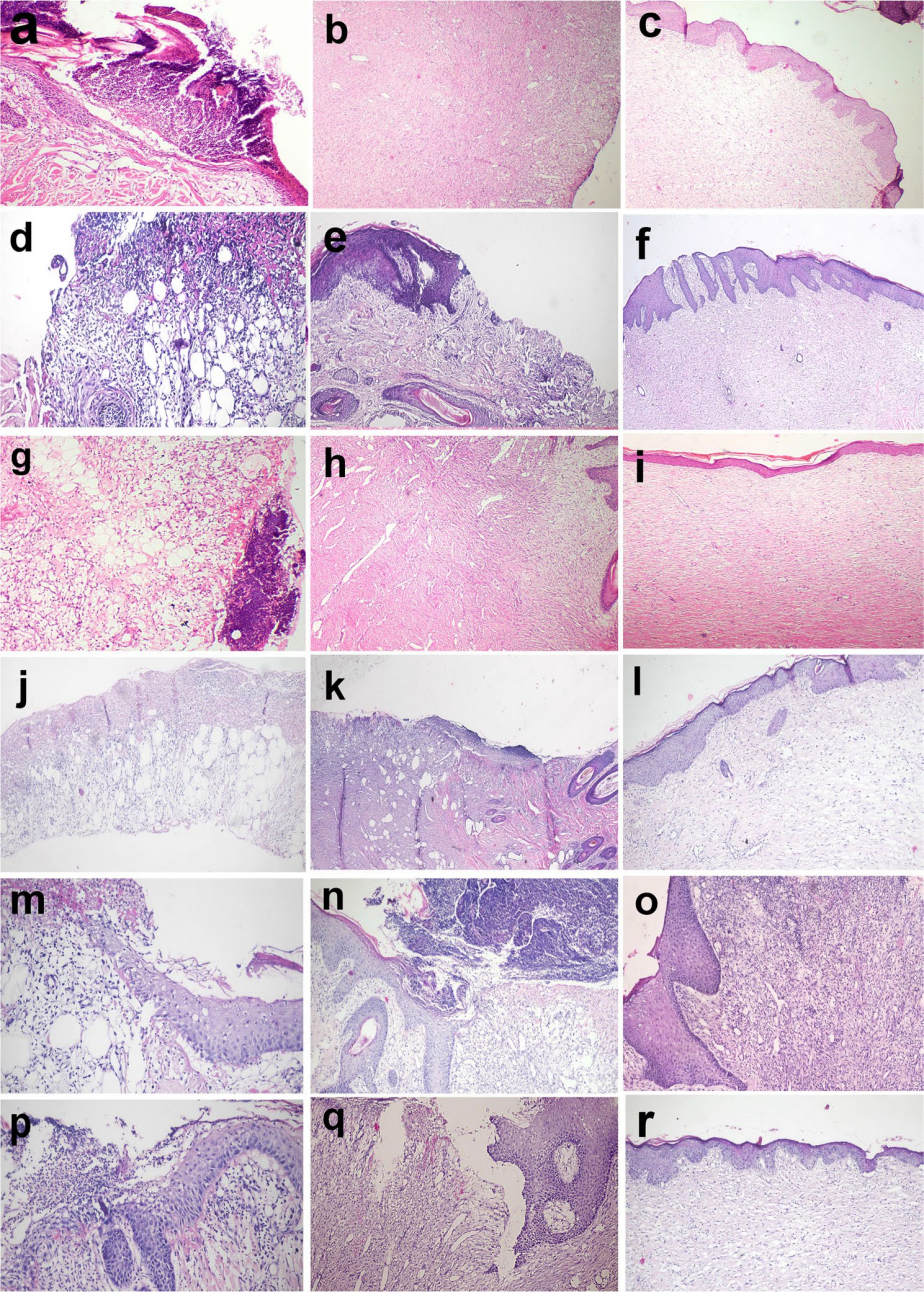
Histopathology of skin wounds in different groups at 5 (X200), 10 (X100) and 20 days (X100) post-induction in dogs. **a**-**c** control wound, (**d**-**f**) Lanolin treated wound, (**g**-**i**) PRP treated wound, (**j**-**l**) PRP- Lanolin treated wounds, (**m**-**o**) g-C3N4 NPs treated wound, (**p**-**r**) PRP- g-C3N4 NPs treated wound (H and E stain)

At 5 days after wound induction in all groups, there was edema and polymorphnuclear leukocytes infiltration with cast formation at the top of the wound. At 10 days after wound induction, subsided inflammation, hyperplasia of epithelium at the periphery of the wound and re-epithelization were observed in wounds of PRP, lanolin and PRP- g-C3N4 NPs groups. Granulation tissue was formed and organized espicially in control, PRP, and lanolin groups. At 20 days after wound induction, the epithelium bridged the wounds in all groups and the granulation tissue was well arranged and organised in PRP, lanolin, PRP-lanolin and PRP- g-C3N4 NPs groups as shown in Fig. 3(a-r).

**Fig. 4:**
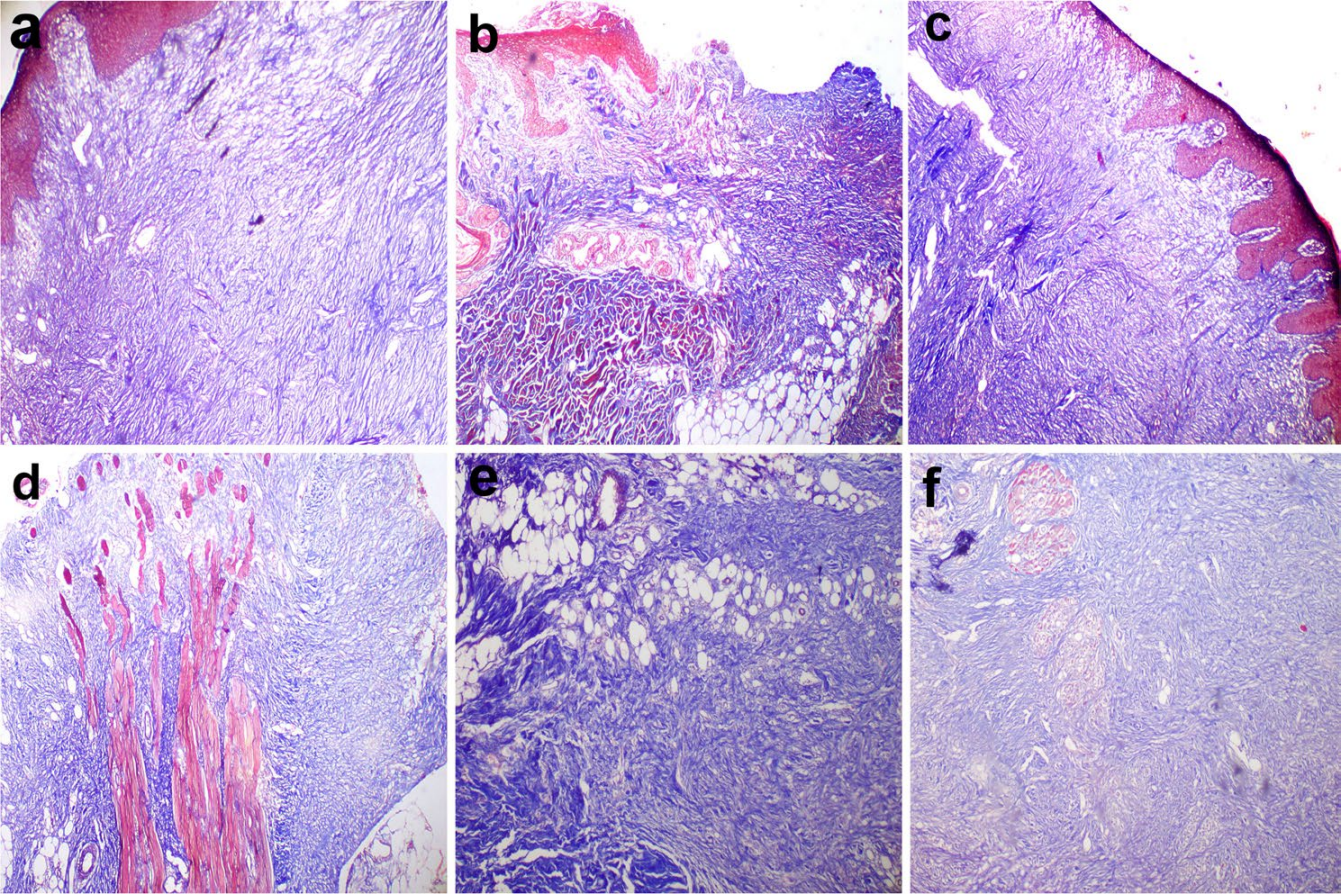
Skin wounds stained with Masson’s Trichrome in different groups at 10 days post-induction. **a** The collagen bundles were disorganized with variation of color and infiltration in the control wound. **b** collagen bundles showed variations of color and breaks in parallel fibers in Lanolin treated wound. **c** the collagen bundles were organized and had parallel wavy fibers with consistent blue color in PRP-treated wound. **d** the collagen bundles were organized, had parallel wavy fibers and breaks in parallel fibers in PRP- Lanolin treated wound. **e** organized wavy parallel collagen bundles with little breaks in g-C3N4 NPs treated wound. **f** abundant collagen bundles with few breaks in PRP- g-C3N4 NPs treated wound. MTC X 100

**Fig. 5:**
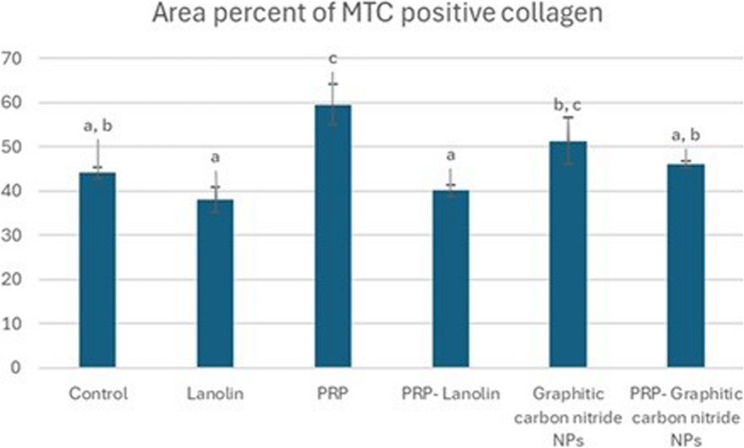
Mean area percent of MTC stained collagen in different groups at 10 days. The columns represent the mean area ± standard error. Columns bearing different lowercase letters are considered significant at *P *value <0.05

The Masson’s Trichrome (MTC) stain highlighted collagen bundles in the skin wound biopsies, appearing blue under microscopic examination. In the control group, the collagen bundles were disorganized, showing color variations and infiltration (Fig. 4a). In contrast, the lanolin-treated group displayed color variations and breaks in the parallel fibers (Fig. 4b). In the PRP-treated wounds, the collagen bundles were well-organized, with parallel wavy fibers and a consistent blue color (Fig. 4c). The PRP- lanolin treated wounds had organized collagen bundles, with parallel wavy fibers but also showed some fiber breaks (Fig. 4d). In g-C₃N₄ NPs -treated wounds, the collagen bundles remained organized, with parallel wavy fibers and minimal breaks (Fig. 4e). Meanwhile, in PRP- g-C₃N₄ NPs treated wounds, abundant collagen bundles with few breaks were observed (Fig. 4f). The mean area percentage of MTC-stained collagen was significantly higher in the PRP-treated wounds compared to the control group, followed by the PRP- g-C₃N₄ NPs treated wounds (Fig. 5).

### Immunohistochemistry

**Fig. 6:**
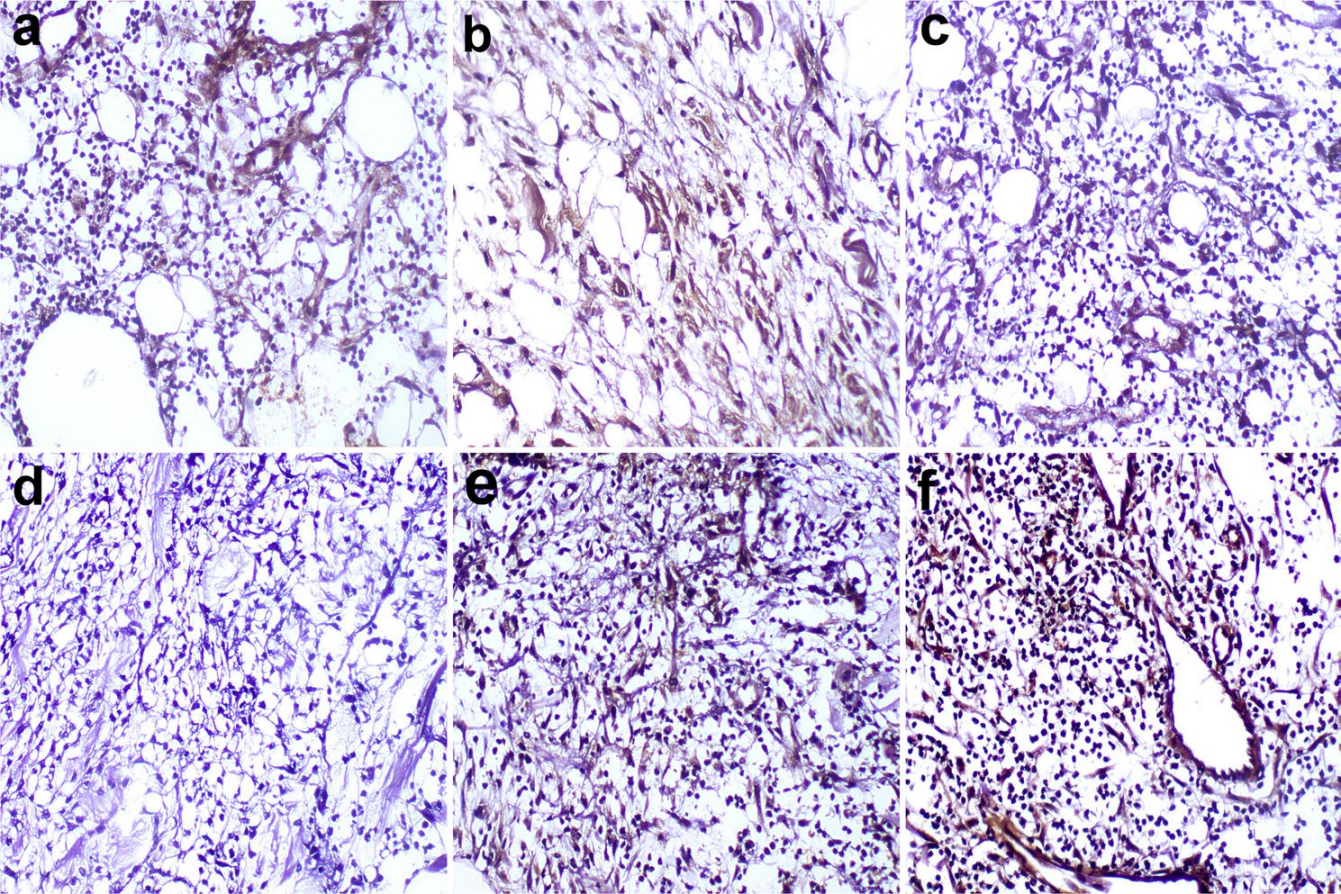
TNF-alpha immunohistochemistry of Skin wounds in different groups at 5 days post-induction. **a** It was mildly to moderately expressed in the control group, **b** moderately to severely expressed in Lanolin-treated wound. **c** moderately to severely expressed in PRP-treated wound, **d** mild to moderately expressed in PRP-lanolin-treated wound. **e** moderately expressed in g-C3N4 NPs treated wound. **f** severely expressed in PRP- g-C3N4 NPs NPs treated wound. Immunoperoxidase X400

**Fig. 7:**
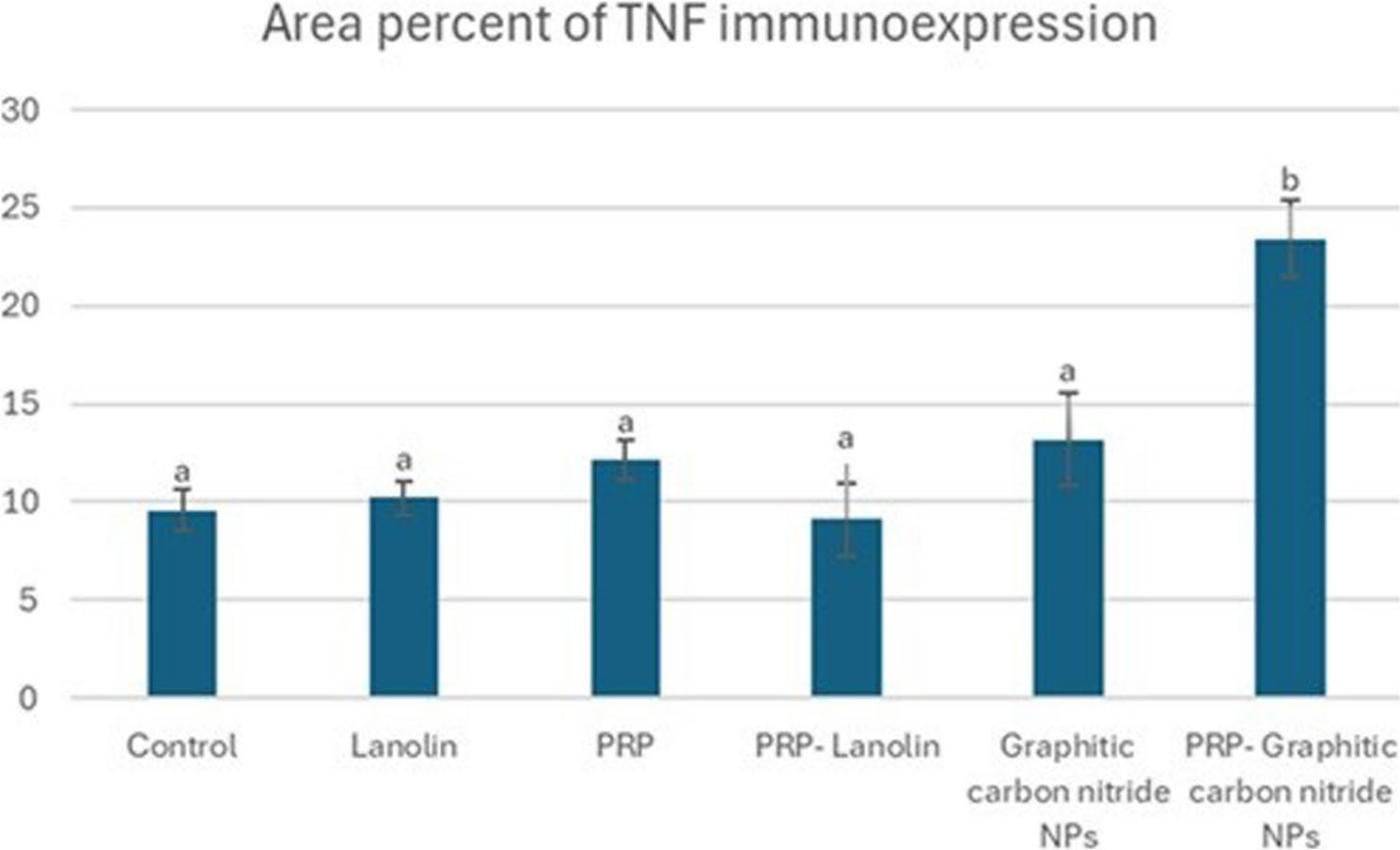
Mean area percentage of TNF-α immunohistochemistry in different groups at 5 days. The columns represent the mean area ± standard error. Columns bearing different lowercase letters are considered significant at *P* value <0.05

TNF-α immuoexpression was observed in the leukocytes infiltrating the wound and endothelial cells lining newly formed blood capillaries. It was mild to moderately expressed in control group (Fig. 6a), moderately to severely expressed in lanolin treated wound (Fig. 6b), moderately to severely expressed in PRP treated wound (Fig. 6c), mild to moderately expressed in PRP- lanolin treated wound (Fig. 6d), mildly expressed in g-C₃N₄ NPs treated wound (Fig. 6e), severely expressed in PRP- g-C3N4 NPs treated wound (Fig. 6f). The mean Area percent of TNF-α immunohistochemistry was significantly elevated in PRP- g-C₃N₄ NPs treated wound compared to control followed by PRP treated wound (Fig. 7).

## Discussion

The current study demonstrates that autologous PRP significantly accelerates wound healing, as evidenced by the highest reduction in wound size and greatest contraction percent across all time points. By day 21, PRP-treated wounds showed the most complete healing, with PRP–g-C3N4 NPs achieving nearly equivalent outcomes. This suggests that g-C3N4 nanoparticles, known for their high surface area and biocompatibility [29], may synergize with or mimic some regenerative properties of PRP.

The moderate performance of PRP–lanolin and lanolin alone further indicates the importance of active components like PRP and nanomaterials in driving effective tissue regeneration. These findings are consistent with prior studies highlighting PRP’s role in enhancing angiogenesis and cellular proliferation in wound sites [30].

The study found that PRP significantly accelerated healing and epithelialization, achieving near-complete wound closure by day 21. Notably, g-C3N4 nanoparticles, both alone and combined with PRP, demonstrated healing percentages and epithelialization rates that closely approached those of PRP. This suggests that g-C3N4 contributes positively to tissue repair. Its known ability to modulate key cellular pathways such as TGF-β, and integrin signaling likely enhances fibroblast proliferation and epithelial cell migration, processes critical to wound healing [31].

SEM analysis revealed that g-C3N4 has a smooth, sheet-like morphology with minimal surface defects, indicating a high surface area ideal for nanoparticle doping. This aligns with previous studies that describe g-C3N4 as forming crumpled or layered nanosheets with good dispersibility and few crevices, supporting its use in biomedical and catalytic applications [29, 32, 33].

Additionally, g-C3N4 large surface area and structural compatibility with biological tissues facilitate cellular attachment and regenerative signaling [11]. Its physicochemical properties and biocompatibility make it a promising scaffold or carrier in tissue repair applications [34]. While PRP drove faster epithelialization in early stages, the similar final epithelial coverage across all groups by day 21 highlights the capacity of g-C3N4 NPs to support and eventually match biological healing performance.

Oxidative stress plays a key role in delaying wound healing, making antioxidant activity a crucial aspect of therapeutic efficacy. This study measured total Antioxidant Capacity (TAC) and malondialdehyde (MDA) a marker of lipid peroxidation to evaluate redox dynamics during wound healing across various treatment groups [35].

PRP-treated animals showed the lowest TAC, indicating high oxidative stress (0.32 ± 0.02), while the control group showed the highest (1.89 ± 0.11). Nano formulations like PRP–g-C₃N₄ NPs (0.90 ± 0.08) and g-C₃N₄ NPs (0.78 ± 0.15) exhibited intermediate TAC, suggesting partial oxidative protection. These lower TAC values suggest higher antioxidant consumption at the wound site, reflecting active scavenging of reactive oxygen species (ROS) generated during early tissue remodeling [36, 37].

This trend persisted through days 10 and 20. PRP consistently showed the lowest TAC (0.01 ± 0.01 on day 10; 0.20 ± 0.017 on day 20), while PRP–g-C₃N₄ NPs preserved significantly higher TAC levels (0.58 ± 0.26 and 0.28 ± 0.03, respectively), indicating better oxidative balance. These findings align with recent studies showing that g-C₃N₄ nanostructures exhibit photocatalytic and antioxidant activity, mitigating oxidative stress during wound healing, and [16, 38, 39].

MDA is a biomarker of lipid peroxidation and oxidative stress. Assessment of tissue malondialdehyde (MDA) revealed that elevated MDA levels indicate increased oxidative stress, which can impair tissue regeneration and delay healing [35]. Reducing MDA levels is critical, as oxidative stress can delay healing by damaging cell membranes, proteins, and DNA [40]. By day 5, the control group displayed the highest MDA (37.22 ± 1.69), while PRP–g-C₃N₄ NPs (5.17 ± 0.91) and g-C₃N₄ NPs (1.37 ± 0.21) showed the most substantial reductions, indicating strong suppression of lipid peroxidation and oxidative stress.

This pattern continued on days 10 and 20. PRP–g-C₃N₄ NPs maintained the lowest MDA (1.23 ± 0.11 on day 10 and 0.06 ± 0.05 on day 20), suggesting sustained antioxidant defense. These outcomes are corroborated by research showing that g-C₃N₄ enhances redox homeostasis and protects against oxidative injury via ROS regulation and photodynamic mechanisms [41–43].

The matrix extracellular phosphoglycoprotein (MEPE) is one of the “matricellular protein” related to SIBLING family (small integrin-binding ligand, N-linked glycoproteins family**)** [44, 45]. Though they can attach to structural ECM elements like collagen fibrils or basement membrane, matricellular proteins are released into the extracellular matrix and are thought to have no role in their mechanical activities [46]. In addition, it is strictly controlled to fine-tune its roles during tissue healing and maintenance [47].

Transforming Growth Factor Beta (TGF-β) is a pivotal cytokine in the wound healing cascade, known for regulating inflammation, stimulating fibroblast activity, and promoting extracellular matrix deposition [48].

The gene expression results across MEPE, TGF-β, and TNF-α suggest a complex interplay between treatment types and timepoints, with platelet-rich plasma (PRP) and its combination with graphitic carbon nitride (g-C₃N₄) nanoparticles showing distinct patterns of regenerative and anti-inflammatory signaling. PRP consistently demonstrated the highest expression levels of MEPE and TGF-β, reflecting its potent activation of osteogenic and regenerative pathways. This is supported by previous findings that highlight PRP’s capability to stimulate TGF-β signaling in wound healing scenarios [49–51]. Graphitic carbon nitride has also been shown to influence TGF-β pathways and oxidative stress responses relevant to fibroblast activity [52].

Interestingly, the PRP and PRP–g-C₃N₄ NP combination also achieved elevated MEPE and TGF-β expression, especially at later time points, while reducing TNF-α levels, consistent with g-C₃N₄’s modulation of inflammatory signaling and redox-sensitive gene networks [31].

Additionally, TNF-α expression was most effectively suppressed in PRP-based treatments, particularly PRP–lanolin by day 10, highlighting the anti-inflammatory strength of platelet-derived factors [53]. This aligns with prior research demonstrating TNF-α suppression in fibroblasts through TGF-β interactions and PRP signaling [54].

The elevated PDGFβ levels observed in the PRP and PRP–g-C₃N₄ NP groups underscore the potent regenerative capacity of platelet-derived therapies, consistent with their known enrichment in growth factors that drive angiogenesis and tissue repair [30]. Graphitic carbon nitride (g-C₃N₄), while biologically inert in isolation, appears to support PRP-mediated growth factor expression, possibly by offering a biocompatible scaffold that enhances local retention and bioactivity of PRP constituents. Recent studies support this synergistic role; g-C₃N₄-based wound dressings have been shown to accelerate healing by enhancing fibroblast proliferation and modulating the local microenvironment [16]. Additionally, g-C₃N₄ composites have demonstrated effective antibacterial activity and photodynamic properties that indirectly support healing by reducing microbial interference [39]. Although g-C₃N₄ alone did not stimulate PDGFβ release, its integration into a PRP-based system yielded a composite that sustained high PDGFβ levels over time, likely by prolonging PRP bioactivity in the wound environment [52]. These findings highlight the potential of combining biological and nanomaterial-based therapies to optimize wound healing outcomes.

Histopathological evaluation revealed that wounds treated with PRP, lanolin, and especially PRP combined with g-C₃N₄ nanoparticles showed progressive healing stages including reduced inflammation, enhanced re-epithelialization, and organized collagen deposition. Masson’s Trichrome staining confirmed that collagen bundles were better aligned and more abundant in PRP and PRP-g-C₃N₄ NPs groups, suggesting a stronger extracellular matrix. These observations are supported by studies showing that PRP can accelerate healing by promoting collagen synthesis and modulating inflammation [55], while g-C₃N₄-based materials have demonstrated anti-inflammatory and pro-collagen effects in infected wounds [56].

Immunohistochemistry further showed variations in TNF-α expression across groups, with PRP-g-C₃N₄ NPs-treated wounds exhibiting elevated TNF-α levels, possibly reflecting an early pro-inflammatory surge necessary for initiating tissue remodeling. Together, these findings underscore the synergistic role of PRP and g-C₃N₄ in enhancing wound healing through both cellular and molecular mechanisms.

Histologically, g-C₃N₄-treated wounds exhibited more organized collagen deposition, improved epithelial architecture, and reduced inflammation. Studies have shown that g-C₃N₄ integrated into scaffolds or nanofibers promotes fibroblast viability, epithelial cell migration, and downregulation of inflammatory cytokines such as TNF-α and IL-6 [57, 58].

The use of graphitic carbon nitride (g-C₃N₄) nanoparticles in our study significantly enhanced cutaneous wound healing in dogs, as evidenced by faster wound contraction, earlier epithelialization, and improved histological organization compared to the control group. These findings align with studies suggesting that g-C₃N₄, owing to its high surface area and two-dimensional layered structure, promotes cellular proliferation and tissue regeneration. Although most prior research has focused on the role of g-C₃N₄ in bone tissue engineering, such as enhancing collagen synthesis and extracellular matrix organization in rabbit bone models [59].

Mechanistically, g-C₃N₄ appears to exert its therapeutic effects through a combination of structural support and bioactivity. Its photocatalytic properties can generate reactive oxygen species (ROS), which help reduce bacterial load while stimulating local immune modulation [60]. demonstrated that g-C₃N₄-based hydrogels reduced inflammation and accelerated healing by inducing macrophage polarization from the pro-inflammatory M1 phenotype to the pro-reparative M2 phenotype. These effects were echoed in our study, where g-C₃N₄-treated wounds showed reduced inflammatory cell infiltration and increased fibroblast density by day 10.

Although there are currently no published studies specifically combining graphitic carbon nitride (g-C₃N₄) nanoparticles with platelet-rich plasma (PRP) for wound healing, related research strongly supports the potential synergy of PRP with nanomaterials. For instance, a study incorporated PRP with titanium dioxide-based nanocomposites (P25/SWCNT/Ag and P25/rGO/Ag) into a gelatin scaffold, which significantly enhanced wound healing outcomes by promoting growth factor retention and antimicrobial effects [57]. Similarly, the combination of selenium nanoparticles with PRP in diabetic mice improved healing speed and tissue regeneration, demonstrating a synergistic effect due to the antioxidant and growth factor-enriching properties of both agents [61].

Additionally, PRP has been shown to benefit from nanocarrier systems that sustain the release of its growth factors. A notable example involved heparin-conjugated PLGA nanospheres that allowed for extended delivery of PRP-derived PDGFΒ-BB, which accelerated dermal regeneration in a mouse model [62]. These findings collectively suggest that pairing PRP with nanomaterials enhances its bioavailability and regenerative performance. Given g-C₃N₄’s known photoactive and antibacterial capabilities, combining it with PRP presents a promising but unexplored approach for future wound healing therapies.

Despite the promising data, the use of g-C₃N₄ in veterinary medicine particularly for canine cutaneous wounds remains a relatively unexplored area. There is a clear need for species-specific research to evaluate biocompatibility, optimal dosing, and long-term safety in dogs. Nevertheless, current evidence from biomedical and material sciences suggests that g-C₃N₄ offers a powerful platform for developing next-generation wound healing systems that are multifunctional, safe, and cost-effective for veterinary clinical use [63].

### Future directions

While our findings confirm the wound-healing potential of g-C₃N₄ nanoparticles in dogs, several areas warrant further investigation. Future studies should explore optimal dosing, long-term safety, and systemic absorption to ensure clinical viability. Special attention should be given to tissue accumulation and potential adverse effects with repeated application.

Another important avenue involves enhancing g-C₃N₄ activity through external stimuli. Light-activated systems, especially those using visible or near-infrared (NIR) light, have shown promise in improving bacterial clearance and angiogenesis [64]. Developing wearable light-based dressings integrated with g-C₃N₄ could expand its therapeutic scope in veterinary medicine.

Additionally, combining g-C₃N₄ with biological agents such as PRP, stem cells, or growth factor-loaded hydrogels may offer synergistic healing benefits by uniting structural, regenerative, and immunomodulatory functions. Investigating controlled-release platforms that allow synchronized delivery of both nanomaterials and biological agents will be essential.

Finally, future efforts should aim to translate g-C₃N₄-based formulations into clinically applicable products, including sprays, ointments, and bioactive dressings. These formulations should be tested across a wider variety of wound types, species, and clinical settings, while considering regulatory pathways, cost-effectiveness, and usability in real-world veterinary practice.

## Conclusion

The present study demonstrated that both PRP and PRP–g-C₃N₄ NPs enhanced the healing of surgically induced cutaneous wounds in dogs through distinct biological effects. PRP alone produced the strongest regenerative response, as demonstrated by faster wound healing, enhanced epithelialization, improved collagen organization, and greater expression of TGF-β, PDGF-β, and MEPE. These findings indicate that a single local PRP treatment appears to be sufficient for promoting the healing of uncomplicated surgical or fresh wounds under the conditions of the present study.

The PRP–g-C₃N₄ NPs treatment provided substantial regenerative effects together with improved redox balance, as indicated by lower lipid peroxidation and greater preservation of total antioxidant capacity. Therefore, the combined treatment may be particularly relevant when wound healing is complicated by excessive oxidative stress, persistent inflammation, or microbial contamination. Considering the reported antimicrobial and antibiofilm properties of g-C₃N₄, this combination may also have potential application in infected or chronic wounds associated with biofilm-forming bacteria. However, this possibility was not directly investigated in the present study and should be confirmed in future studies using appropriate infected and chronic wound models.

## Data Availability

All data is available based on a reasonable request.

## Abbreviations

g-C3N4: Graphitic carbon nitride nanoparticles
IL-6: Interleukin-6
MDA: Malondialdehyde
MEPE: Matrix extracellular phosphoglycoprotien
PDGFβ: Platelet derived growth factor Beta
PRP: Platelet Rich Plasma
TAC: Total antioxidant capacity
TGF-β: Transforming growth factor beta
TNF-α: Tumor necrosis factor alpha

## References

[1] Bohling MW, Henderson RA. Differences in cutaneous wound healing between dogs and cats. Vet Clin Small Anim Pract. 2006;36(4):687–92. 10.1016/j.cvsm.2006.02.001 16787783

[2] Abu-Seida AM. Effect of propolis on experimental cutaneous wound healing in dogs. Veterinary Med Int. 2015;2015(1):672643. 10.1155/2015/672643 PMC4691486 26783495

[3] Iacopetti I, Patruno M, Melotti L, Martinello T, Bedin S, Badon T, et al. Autologous platelet-rich plasma enhances the healing of large cutaneous wounds in dogs. Front Vet Sci. 2020;7:575449. 33195571 10.3389/fvets.2020.575449 PMC7649378

[4] Hussein SM. Effects of autologous platelet-rich plasma on skin healing in dogs. Iraqi J Vet Sci. 2018;32(2):275–83.

[5] Wafy MN, Hassan EA, Saeed S, Khattab MS, AbuBakr HO, Yassin AM, Abu-Seida AM. Nanostructured propolis ointment and platelet-rich plasma as novel biotherapeutics for cutaneous wound repair in an experimental canine model. Discover Nano. 2026;21(1):80. 41865140 10.1186/s11671-026-04482-0 PMC13005797

[6] Jee CH, Eom NY, Jang HM, Jung HW, Choi ES, Won JH, et al. Effect of autologous platelet-rich plasma application on cutaneous wound healing in dogs. J Vet Sci. 2016;17(1):79–87. 27051343 10.4142/jvs.2016.17.1.79 PMC4808647

[7] Farghali HA, AbdElKader NA, Khattab MS, AbuBakr HO. Evaluation of subcutaneous infiltration of autologous platelet-rich plasma on skin-wound healing in dogs. Biosci Rep. 2017;37(2):BSR20160503. 28246352 10.1042/BSR20160503 PMC5469334

[8] Kalashnikova I, Das S, Seal S. Nanomaterials for wound healing: scope and advancement. Nanomedicine. 2015;10(16):2593–612. 26295361 10.2217/NNM.15.82

[9] Wafy MN, Hassan EA, Saeed S, Khattab MS, AbuBakr HO, Abu-Seida AM. Therapeutic Efficacy of Zinc Oxide Nanoparticles Ointment in Promoting Wound Healing in Dogs: A Clinical Study. J Appl Veterinary Sci. 2025;10(3):24–33.

[10] Alencar MSF, Silva GHC, da Silva AA, de Azevedo AKR, Pereira MA, do Nascimento JCC, et al. Evidence-based practices on nanotechnology in wound treatment. Cad Pedagogico. 2024;21(12):e10234.

[11] Joy J, George E, Vijayan PP, Anas S, Thomas S. An overview of synthesis, morphology, and versatile applications of nanostructured graphitic carbon nitride (g-C3N4). J Ind Eng Chem. 2024;133:74–89.

[12] Alwin E, Zieliński M, Suchora A, Gulaczyk I, Piskuła Z, Pietrowski M. High surface area, spongy graphitic carbon nitride derived by selective etching by Pt and Ru nanoparticles in hydrogen. J Mater Sci. 2022;57(33):15705–21.

[13] Cheng N, Tian J, Liu Q, Ge C, Qusti AH, Asiri AM, et al. Au-nanoparticle-loaded graphitic carbon nitride nanosheets: green photocatalytic synthesis and application toward the degradation of organic pollutants. ACS Appl Mater Interfaces. 2013;5(15):6815–9. 23875941 10.1021/am401802r

[14] Ladva SA, Travis W, Quesada-Cabrera R, Rosillo-Lopez M, Afandi A, Li Y, et al. Nanoscale, conformal films of graphitic carbon nitride deposited at room temperature: a method for construction of heterojunction devices. Nanoscale. 2017;9(43):16586–90. 29072750 10.1039/c7nr06489f

[15] Cui H, Gu Z, Chen X, Lin L, Wang Z, Dai X, et al. Stimulating antibacterial activities of graphitic carbon nitride nanosheets with plasma treatment. Nanoscale. 2019;11(39):18416–25. 31576862 10.1039/c9nr03797g

[16] Nemati D, Ashjari M, Rashedi H, Yazdian F, Navaei-Nigjeh M. PVA-based nanofiber containing cellulose modified with graphitic carbon nitride/nettles/trachyspermum accelerates wound healing. Biotechnol Prog. 2021;37(6):e3200. 34346569 10.1002/btpr.3200

[17] Wilkinson HN, Hardman MJ. Wound healing: cellular mechanisms and pathological outcomes. Open Biol. 2020;10(9):200223. 32993416 10.1098/rsob.200223 PMC7536089

[18] Li X, Zhang J, Shen L, Ma Y, Lei W, Cui Q, Zou G. Preparation and characterization of graphitic carbon nitride through pyrolysis of melamine. Appl Phys A. 2009;94:387–92.

[19] Wafy MN, Hassan EA, Saeed S, Khattab MS, AbuBakr HO, Abu-Seida AM. The effects of platelet rich plasma and zinc oxide nanoparticle on skin wound healing in dogs. Sci Rep. 2026;16(1):16986. 42230869 10.1038/s41598-026-54633-7 PMC13230633

[20] Mansoub NH, Gürdal M, Karadadaş E, Kabadayi H, Vatansever S, Ercan G. The role of PRP and adipose tissue-derived keratinocytes on burn wound healing in diabetic rats. BioImpacts. 2017;8(1):5. 29713597 10.15171/bi.2018.02 PMC5915708

[21] Sardari K, Pedram S, Zojaji V, Maleki M, Mohri M, Dehgan M, et al. Effects of Zn-7^®^ on open wound healing in dogs. Comp Clin Pathol. 2006;15:237–43.

[22] Sardari K, Kazemi H, Emami MR, Movasaghi AR, Goli AA. Role of collagen cross-linking on equine wound contraction and healing. Comp Clin Pathol. 2009;18:239–47.

[23] Soares CS, Dias IR, Pires MA, Carvalho PP. Canine-origin platelet-rich fibrin as an effective biomaterial for wound healing in domestic cats: a preliminary study. Vet Sci. 2021;8(10):213. 34679043 10.3390/vetsci8100213 PMC8539014

[24] Bradford MM. A rapid and sensitive method for the quantitation of microgram quantities of protein utilizing the principle of protein-dye binding. Anal Biochem. 1976;72(1–2):248–54. 942051 10.1016/0003-2697(76)90527-3

[25] Koracevic D, Koracevic G, Djordjevic V, Andrejevic S, Cosic V. Method for the measurement of antioxidant activity in human fluids. J Clin Pathol. 2001;54(5):356–61. 11328833 10.1136/jcp.54.5.356 PMC1731414

[26] Ohkawa H, Ohishi N, Yagi K. Assay for lipid peroxides in animal tissues by thiobarbituric acid reaction. Anal Biochem. 1979;95(2):351–8. 36810 10.1016/0003-2697(79)90738-3

[27] Livak KJ, Schmittgen TD. Analysis of relative gene expression data using real-time quantitative PCR and the 2 – ∆∆CT method. Methods. 2001;25(4):402–8. 11846609 10.1006/meth.2001.1262

[28] Yan X, Su X. Linear regression analysis: theory and computing. Singapore: World Scientific; 2009.

[29] Abbas S, Khurshid H, Irfan M, Aamir M. Characterisation analysis of sulphur and phosphorous doped and co-doped graphitic carbon nitride (g-C3N4) materials. Lond J Phys. 2024;1(2):1–11.

[30] Oneto P, Etulain J. PRP in wound healing applications. Platelets. 2021;32(2):189–99. 33251921 10.1080/09537104.2020.1849605

[31] Amit C, Sathe G, Shunmugam A, Athyala PK, Ghose V, Chitipothu S, et al. Graphitic carbon nitride causes widespread global molecular changes in epithelial and fibroblast cells. ACS Omega. 2021;6(14):9368–80. 33869917 10.1021/acsomega.0c05513 PMC8047657

[32] Ma TY, Tang Y, Dai S, Qiao SZ. Proton-functionalized two-dimensional graphitic carbon nitride nanosheet: an excellent metal-/label-free biosensing platform. Small. 2014;10(12):2382–9. 24596304 10.1002/smll.201303827

[33] Xu J, Li D, Chen Y, Tan L, Kou B, Wan F, et al. Constructing sheet-on-sheet structured graphitic carbon nitride/reduced graphene oxide/layered MnO2 ternary nanocomposite with outstanding catalytic properties on thermal decomposition of ammonium perchlorate. Nanomaterials. 2017;7(12):450. 29244721 10.3390/nano7120450 PMC5746940

[34] Fei J, Peng X, Jiang L, Yuan X, Chen X, Zhao Y, Zhang W. Recent advances in graphitic carbon nitride as a catalyst for heterogeneous Fenton-like reactions. Dalton Trans. 2021;50(46):16887–908. 34734599 10.1039/d1dt02367e

[35] Abdelgalil AI, Yassin AM, Khattab MS, Abdelnaby EA, Marouf SA, Farghali HA, et al. Platelet-rich plasma attenuates the UPEC-induced cystitis via inhibiting MMP-2, 9 activities and downregulation of NGF and VEGF in Canis lupus familiaris model. Sci Rep. 2024;14(1):13612. 38871929 10.1038/s41598-024-63760-y PMC11176177

[36] Asif M, Chaudhry AS, Ashar A, Rashid HB, Saleem MH, Aslam HB, Aziz A. Zinc oxide nanoparticles accelerate the healing of methicillin-resistant Staphylococcus aureus (MRSA)-infected wounds in rabbits. Asian Pac J Trop Biomed. 2023;13(11):488–96.

[37] Moalwi A, Naik K, Muddapur UM, Aldoah B, AlWadai HH, Alamri AM et al. Harnessing the power of Saussurea obvallata zinc oxide nanoparticles for accelerated wound healing and antimicrobial action. Int J Nanomed 2024:13071–94. 10.2147/IJN.S480891 PMC11627110 39654801

[38] Liu Y, Zhou A, Zhang Y, Tian Z, Cheng X, Gao Y, et al. A photoactive self-healing carboxymethyl chitosan-based hydrogel for accelerated infected wound healing through simultaneously modulating multiple critical tissue repair factors. Int J Biol Macromol. 2023;242:124631. 37116834 10.1016/j.ijbiomac.2023.124631

[39] Wang Y, Xiong J, Xin L, Li Y, Huang H, Miao W. Photosensitizer-synergized g-carbon nitride nanosheets with enhanced photocatalytic activity for eradicating drug-resistant bacteria and promoting wound healing. Chin Chem Lett. 2025;36(4):110003.

[40] Ahmad SU, Binti Aladdin NA, Jamal JA, Shuid AN, Mohamed IN. Evaluation of wound-healing and antioxidant effects of Marantodes pumilum (Blume) Kuntze in an excision wound model. Molecules. 2021;26(1):228. 33466302 10.3390/molecules26010228 PMC7795968

[41] Taheri H, Unal MA, Sevim M, Gurcan C, Ekim O, Ceylan A, et al. Photocatalytically active graphitic carbon nitride as an effective and safe 2D material for in vitro and in vivo photodynamic therapy. Small. 2020;16(10):1904619. 10.1002/smll.201904619 31971659

[42] Rajaji U, Selvi SV, Chen SM, Chinnapaiyan S, Chen TW, Govindasamy M. A nanocomposite consisting of cuprous oxide supported on graphitic carbon nitride nanosheets for non-enzymatic electrochemical sensing of 8-hydroxy-2′-deoxyguanosine. Microchim Acta. 2020;187:1–10. 10.1007/s00604-020-04416-2 32686000

[43] Yadav P, Mimansa, Kailasam K, Shanavas A. Nontoxic metal-free visible light-responsive carbon nitride quantum dots cause oxidative stress and cancer-specific membrane damage. ACS Appl Bio Mater. 2022;5(3):1169–78. 35191305 10.1021/acsabm.1c01219

[44] Fisher LW, Torchia DA, Fohr B, Young MF, Fedarko NS. Flexible structures of SIBLING proteins, bone sialoprotein, and osteopontin. Biochem Biophys Res Commun. 2001;280(2):460–5. 11162539 10.1006/bbrc.2000.4146

[45] Cárdenas-León CG, Mäemets-Allas K, Klaas M, Lagus H, Kankuri E, Jaks V. Matricellular proteins in cutaneous wound healing. Front Cell Dev Biol. 2022;10:1073320. 36506087 10.3389/fcell.2022.1073320 PMC9730256

[46] Feng D, Ngov C, Henley N, Boufaied N, Gerarduzzi C. Characterization of matricellular protein expression signatures in mechanistically diverse mouse models of kidney injury. Sci Rep. 2019;9(1):16736. 31723159 10.1038/s41598-019-52961-5 PMC6854083

[47] Nikoloudaki G, Creber K, Hamilton DW. Wound healing and fibrosis: a contrasting role for periostin in skin and the oral mucosa. Am J Physiol Cell Physiol. 2020;318(6):C1065–77. 32267719 10.1152/ajpcell.00035.2020 PMC7311745

[48] Penn JW, Grobbelaar AO, Rolfe KJ. The role of the TGF-β family in wound healing, burns and scarring: a review. Int J Burns Trauma. 2012;2(1):18. 22928164 PMC3415964

[49] Zambruno G, Marchisio PC, Marconi A, Vaschieri C, Melchiori A, Giannetti A, et al. Transforming growth factor-beta 1 modulates beta 1 and beta 5 integrin receptors and induces the de novo expression of the alpha v beta 6 heterodimer in normal human keratinocytes: implications for wound healing. J Cell Biol. 1995;129(3):853–65. 7537276 10.1083/jcb.129.3.853 PMC2120435

[50] Lyras DN, Kazakos K, Tryfonidis M, Agrogiannis G, Botaitis S, Kokka A, et al. Temporal and spatial expression of TGF-β1 in an Achilles tendon section model after application of platelet-rich plasma. Foot Ankle Surg. 2010;16(3):137–41. 20655014 10.1016/j.fas.2009.09.002

[51] Laidding SR, Josh F, Faruk M, Palissei AS, Satria B, Bukhari A, et al. Combination of platelet-rich plasma and stromal vascular fraction on the level of transforming growth factor-β in rat subjects experiencing deep dermal burn injury. Ann Med Surg. 2020;60:737–42. 10.1016/j.amsu.2020.11.088 PMC7779951 33425344

[52] Jia R, He C, Wang S, Gao Y, Song L, Wang P, et al. Recent advances in graphitic carbon nitride-based heterojunction for biomedical applications. Chem Eng J. 2024;500:157464.

[53] Eom YW, Hong JE, Jung PY, Yoon Y, Yoo SH, Hong J, et al. TGF-β expressed by M2 macrophages promotes wound healing by inhibiting TSG-6 expression by mesenchymal stem cells. PLoS ONE. 2025;20(4):e0316692. 40257993 10.1371/journal.pone.0316692 PMC12011265

[54] Goldberg MT, Han YP, Yan C, Shaw MC, Garner WL. TNF-α suppresses α-smooth muscle actin expression in human dermal fibroblasts: an implication for abnormal wound healing. J Invest Dermatol. 2007;127(11):2645–55. 17554369 10.1038/sj.jid.5700890 PMC2366884

[55] Gad SB, Hafez MH, El-Sayed YS. Platelet-rich plasma and/or sildenafil topical applications accelerate and better repair wound healing in rats through regulation of proinflammatory cytokines and collagen/TGF-β1 pathway. Environ Sci Pollut Res. 2020;27(32):40757–68. 10.1007/s11356-020-10042-5 32671702

[56] Youshi M, Farahpour MR, Tabatabaei ZG. Facile fabrication of carboxymethylcellulose/ZnO/g-C3N4 containing nutmeg extract with photocatalytic performance for infected wound healing. Sci Rep. 2023;13(1):18704. 37907545 10.1038/s41598-023-45921-7 PMC10618236

[57] Deshmukh S, Pawar K, Koli V, Pachfule P. Emerging graphitic carbon nitride-based nanobiomaterials for biological applications. ACS Appl Bio Mater. 2023;6(4):1339–67. 37011107 10.1021/acsabm.2c01016

[58] Wen T, Xiong S, Zhao H, Wang J, Wang C, Long Z, et al. Polylactic acid-based dressing with oxygen generation and enzyme-like activity for accelerating both light-driven biofilm elimination and wound healing. Burns Trauma. 2024;12:tkae041. 39464502 10.1093/burnst/tkae041 PMC11510456

[59] Sadek AA, Abd-Elkareem M, Abdelhamid HN, Moustafa S, Hussein K. Repair of critical-sized bone defects in rabbit femurs using graphitic carbon nitride (g-C3N4) and graphene oxide (GO) nanomaterials. Sci Rep. 2023;13(1):5404. 37012344 10.1038/s41598-023-32487-7 PMC10070441

[60] Tian R, Liu J, Dou G, Lin B, Chen J, Yang G, et al. Synergistic antibiosis with spatiotemporal controllability based on multiple-responsive hydrogel for infectious cutaneous wound healing. Smart Mater Med. 2022;3:304–14.

[61] Karas RA, Alexeree S, Elsayed H, Attia YA. Assessment of wound healing activity in diabetic mice treated with a novel therapeutic combination of selenium nanoparticles and platelets rich plasma. Sci Rep. 2024;14(1):5346. 38438431 10.1038/s41598-024-54064-2 PMC10912747

[62] La WG, Yang HS. Heparin-conjugated poly(lactic-co-glycolic acid) nanospheres enhance large-wound healing by delivering growth factors in platelet-rich plasma. Artif Organs. 2015;39(4):388–94. 25284020 10.1111/aor.12389

[63] Guo H, Huang H, Li Y, Lu S, Xue M, Weng W, et al. Stepwise preparation of Ti-doped functionalized carbon nitride nanoparticles and hybrid TiO₂/graphitic-C₃N₄ for detection of free residual chlorine and visible-light photocatalysis. 2019;55(92):13848–51. 10.1039/c9cc06086c 31670359

[64] Cheng H, Wang N, Yang Y, Jiao X, Han P, Duan W, et al. The enhanced visible light driven photocatalytic activity of zinc porphyrin/g-C3N4 nanosheet for efficient bacterial infected wound healing. J Colloid Interface Sci. 2023;643:183–95. 37058893 10.1016/j.jcis.2023.04.002

